# Supplementary material for: The Accuracy of Semi-Empirical Quantum Chemistry Methods on Soot Formation Simulation
Source: Int J Mol Sci. 2022 Nov 2;23(21):13371. doi: 10.3390/ijms232113371 (PMC9657144; doi:10.3390/ijms232113371)
Supplement: Supplementary file 1 [file ijms-23-13371-s001.zip › ijms-1963104-supplementary.pdf]

Supporting Information of

The Accuracy of Semi-empirical Quantum Chemistry Methods on Soot Formation Simulation

Yang Cong[1], Yu Zhai[1], Xin Chen[2,†], and Hui Li[1,†]

[1] *Institute of Theoretical Chemistry, College of Chemistry, Jilin University, 2519 Jiefang Road, Changchun 130023, Peoples Republic of China*

[2] *Shenzhen Bay Laboratory, Shenzhen 518055, China*

[†]Email: chenxin@szbl.ac.cn; prof_huili@jlu.edu.cn.

1 Energy profiles shape of soot in MD trajectory

In section 2.2 of the article, we performed molecular dynamics and extracted 84 soot structures from the global trajectory. For these structures, their trajectory changes over a time length of about 100-200 fs were recorded, and the Relative energy change curves of each structure during this period of time were calculated using different methods (for example AM1, PM6, PM7, xTB, DFTB).

We classify the trajectories of the above 84 soot structures according to the number of carbon atoms. The naming rule is: "C-XX(number of carbon atoms)-XX (structure number)", if there is one or more oxygen atoms in the structure, add "-O" at the end ". Among all trajectories, the trajectories before the structure named "C-10-7-O" are from the molecular dynamics based on the DFT method, and all the structures after this are from the molecular dynamics based on the reaction force field.

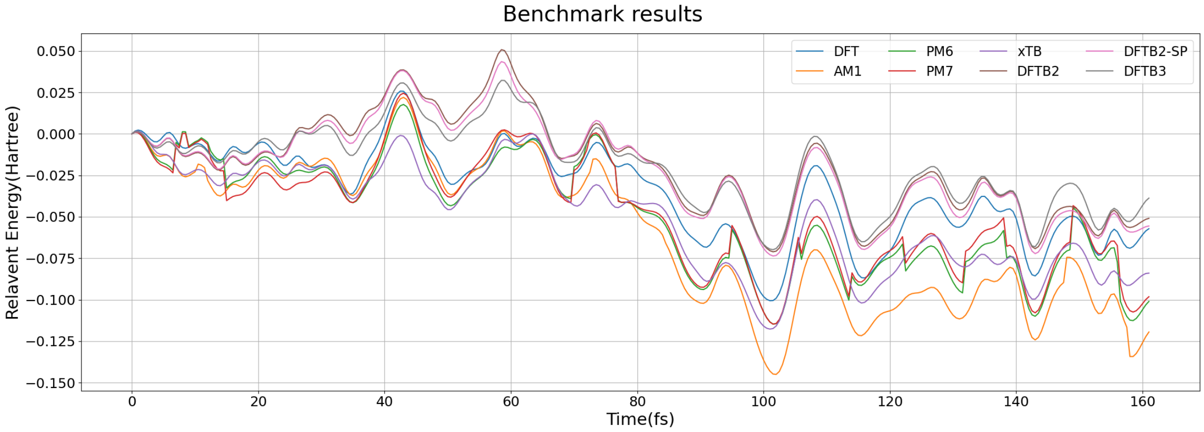

Figure S1: Carbon-4,Trajectory-1.

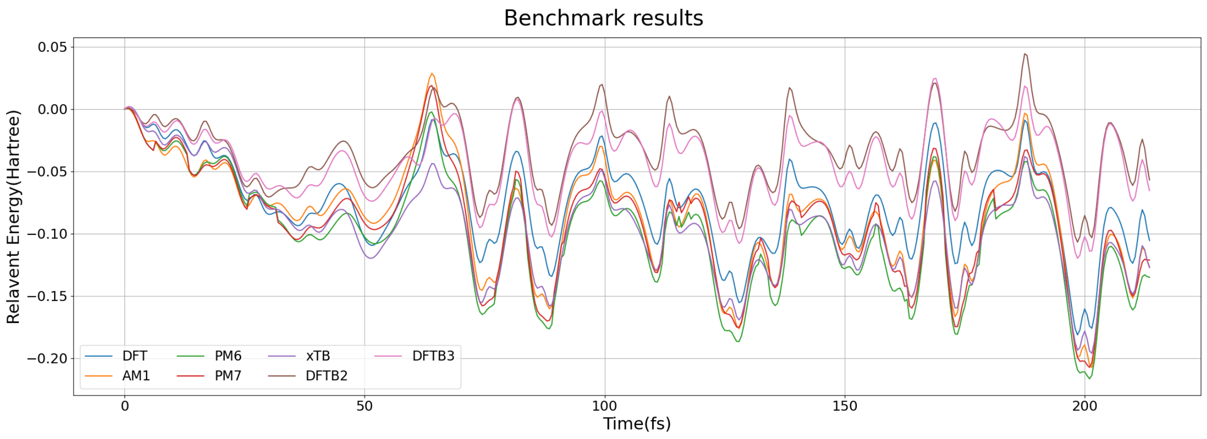

Figure S2: Carbon-4,Trajectory-2.

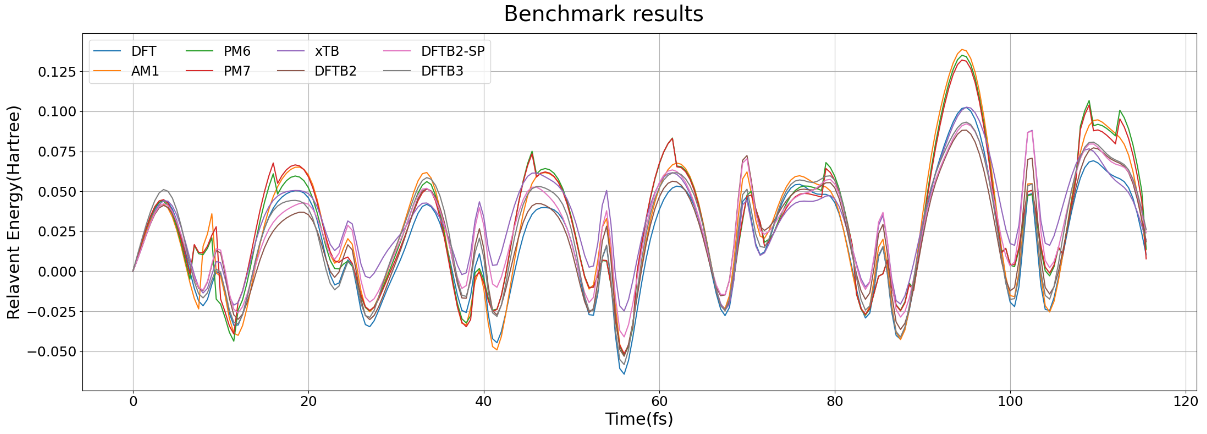

Figure S3: Carbon-5,Trajectory-1.

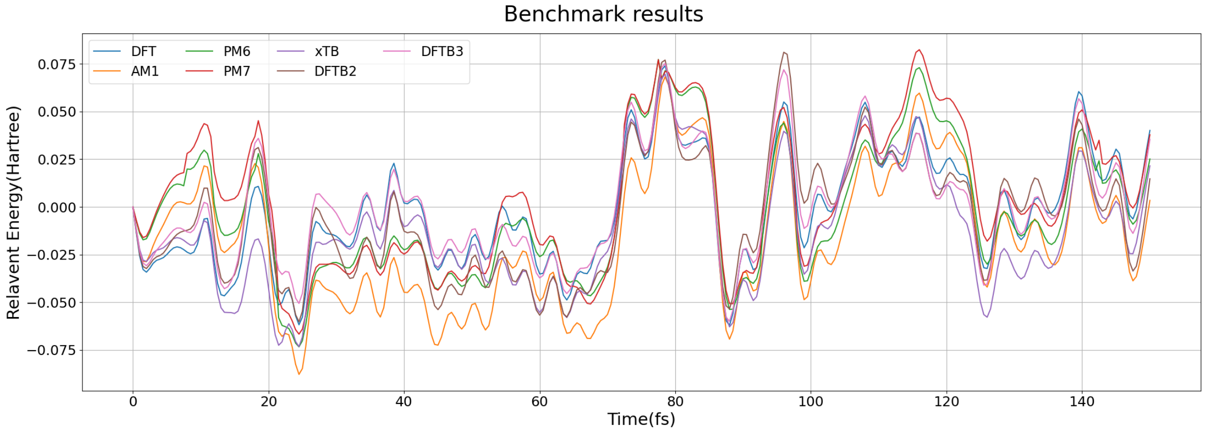

Figure S4: Carbon-5,Trajectory-2.

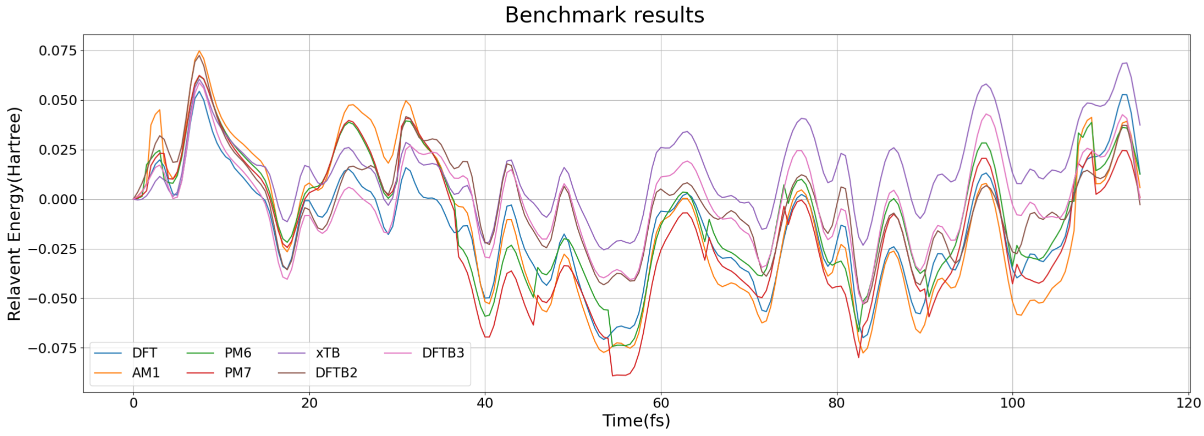

Figure S5: Carbon-5,Trajectory-3.

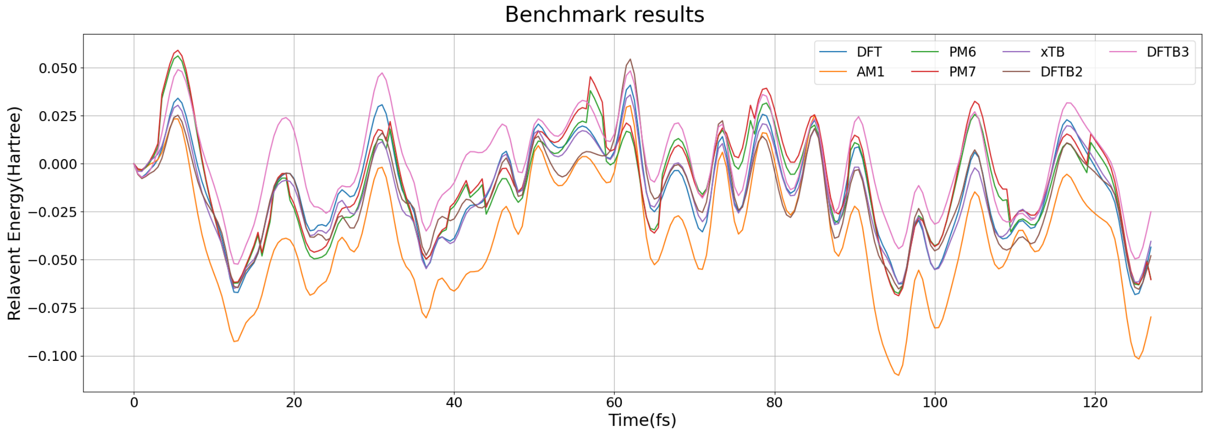

Figure S6: Carbon-5,Trajectory-4.

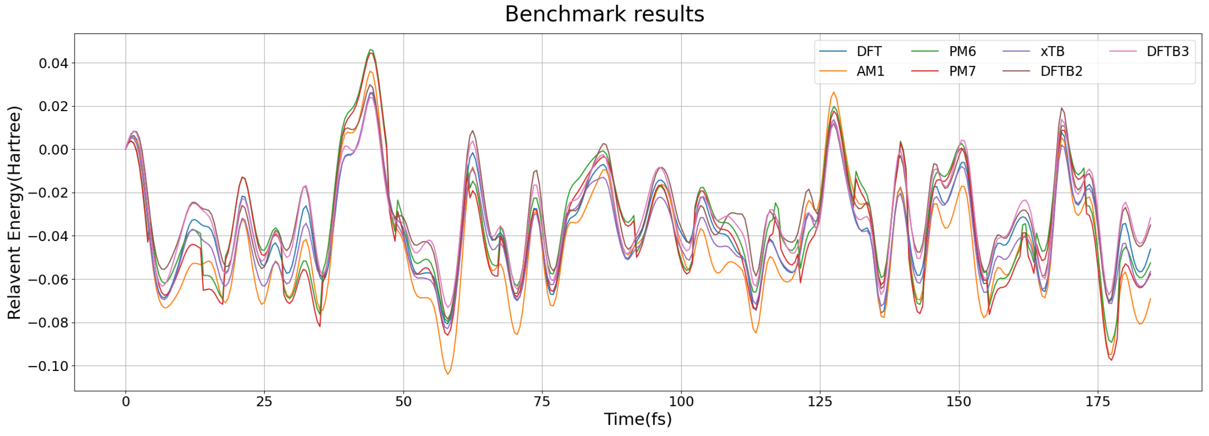

Figure S7: Carbon-5,Trajectory-5.

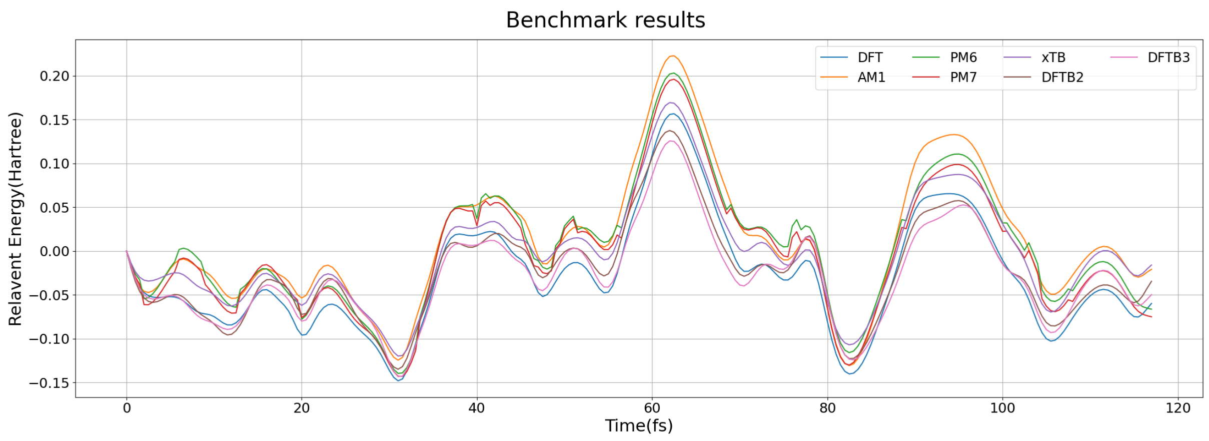

Figure S8: Carbon-5,Trajectory-6.

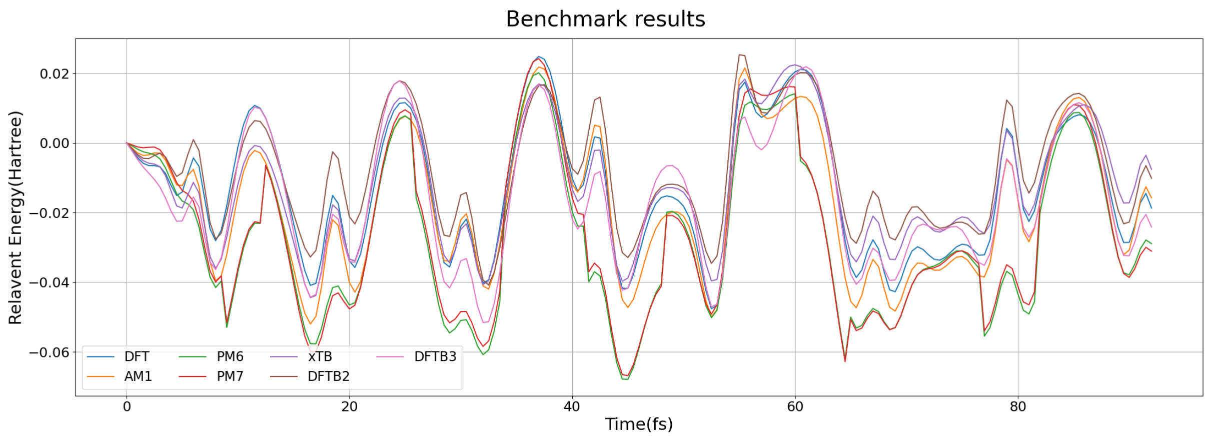

Figure S9: Carbon-5,Trajectory-7.

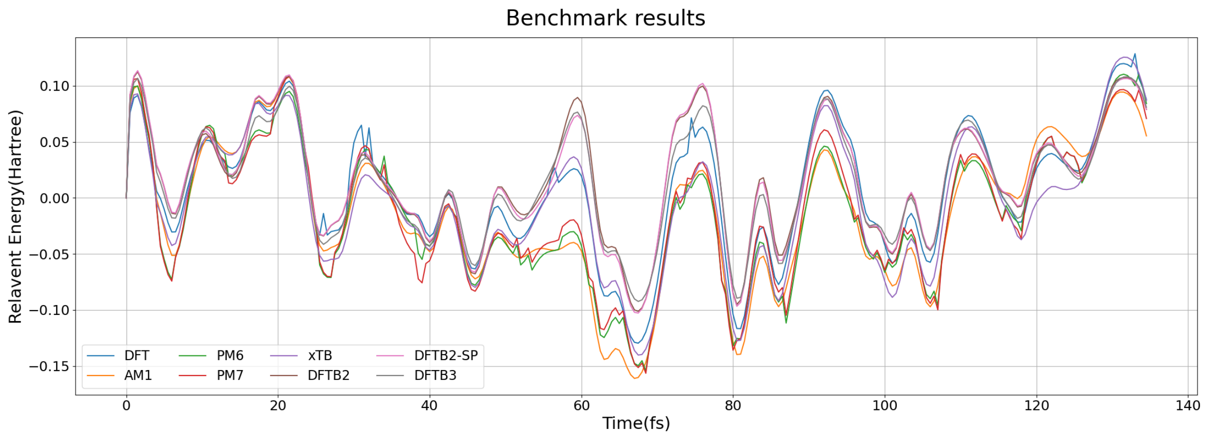

Figure S10: Carbon-6,Trajectory-1.

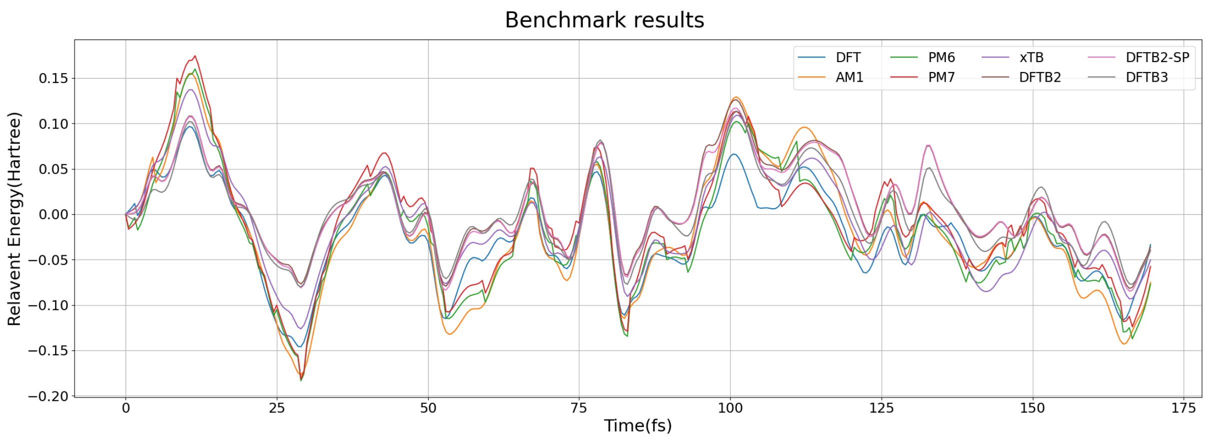

Figure S11: Carbon-6,Trajectory-2.

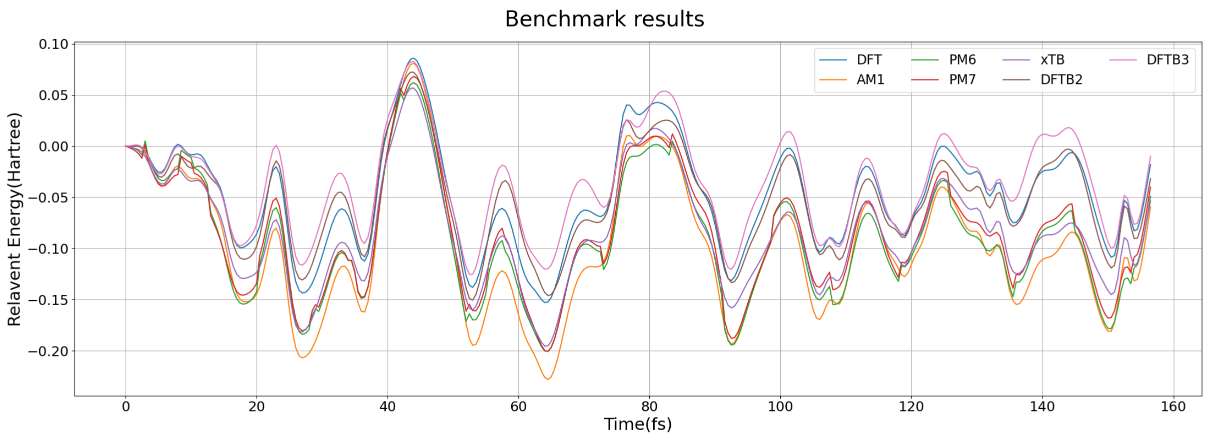

Figure S12: Carbon-6,Trajectory-3.

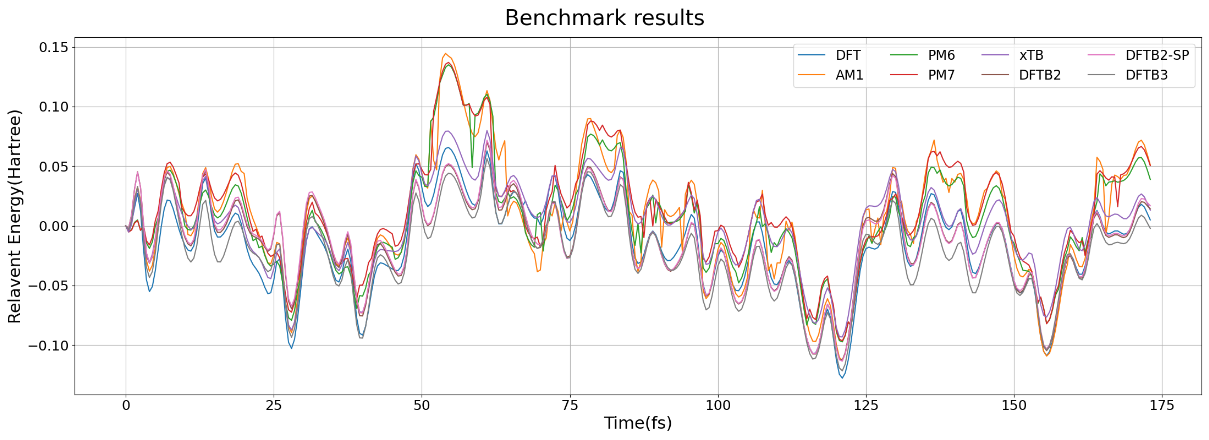

Figure S13: Carbon-6,Trajectory-4.

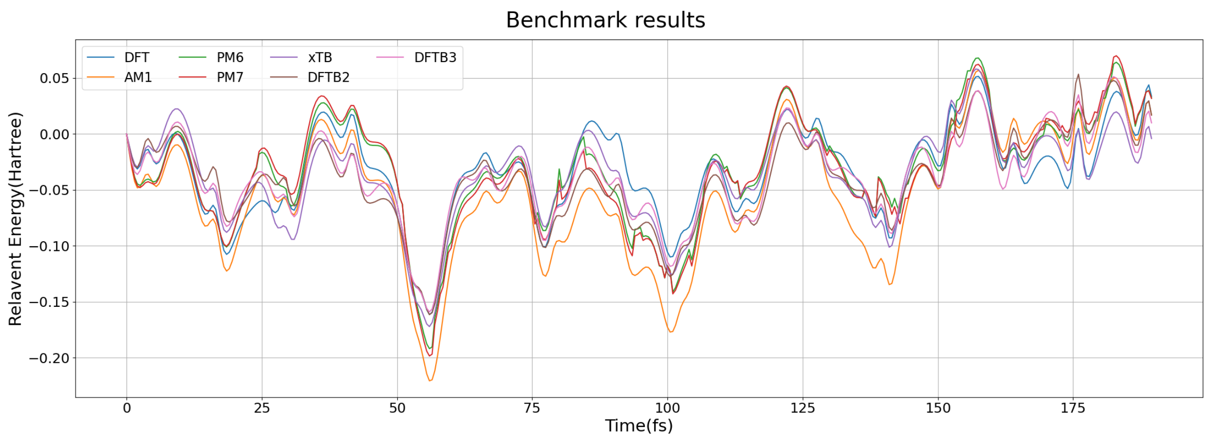

Figure S14: Carbon-6,Trajectory-5.

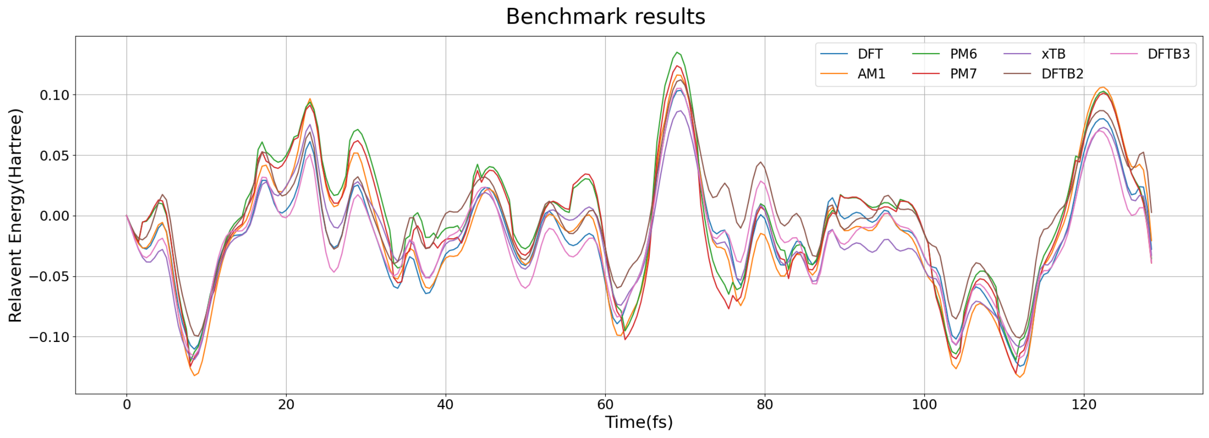

Figure S15: Carbon-6,Trajectory-6.

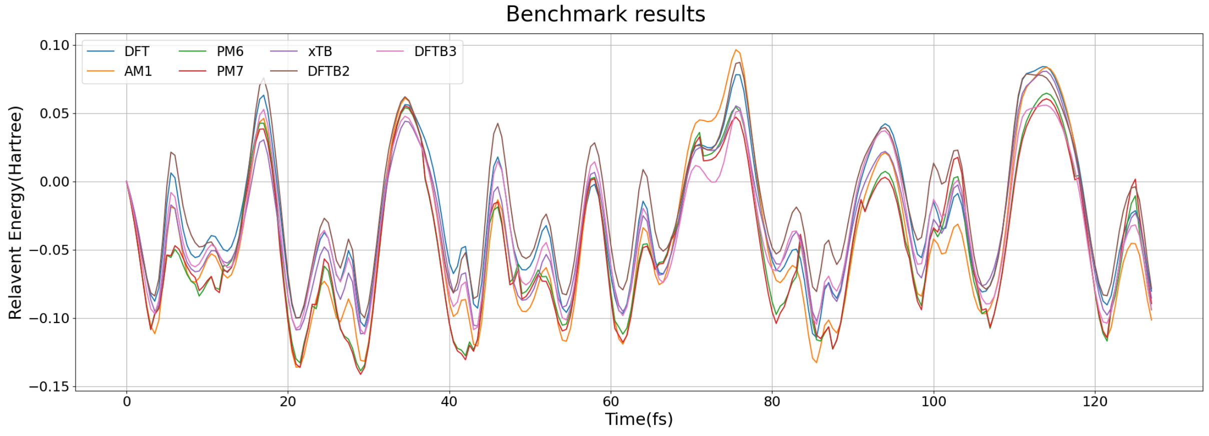

Figure S16: Carbon-6,Trajectory-7.

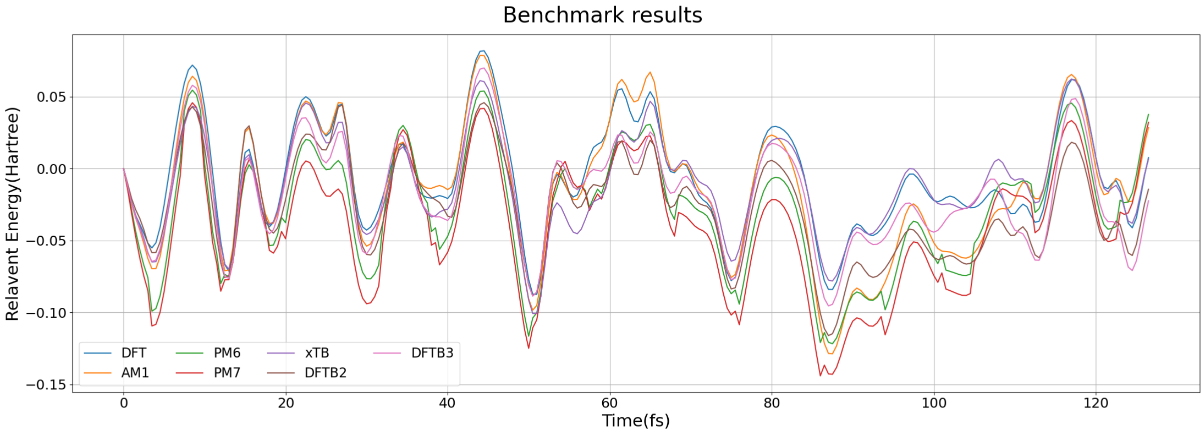

Figure S17: Carbon-6,Trajectory-8.

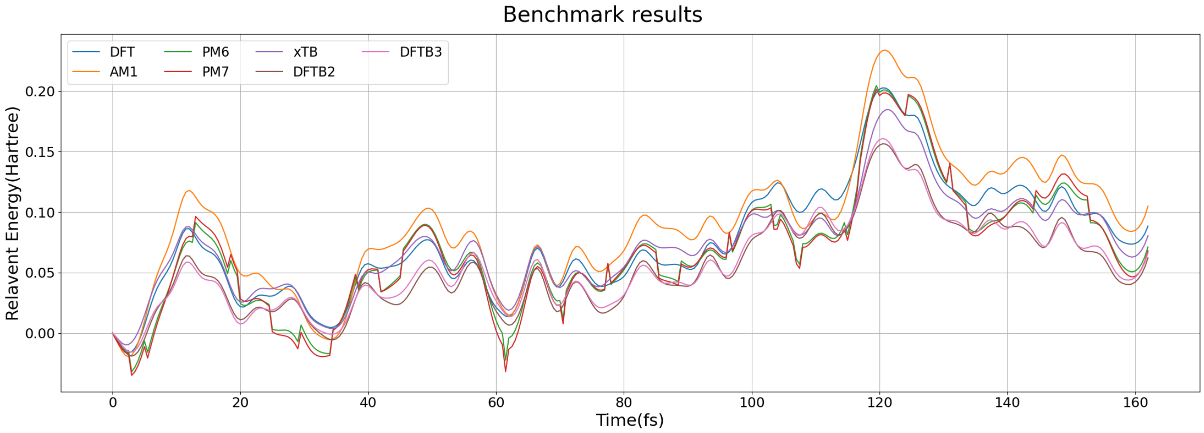

Figure S18: Carbon-6,Trajectory-9.

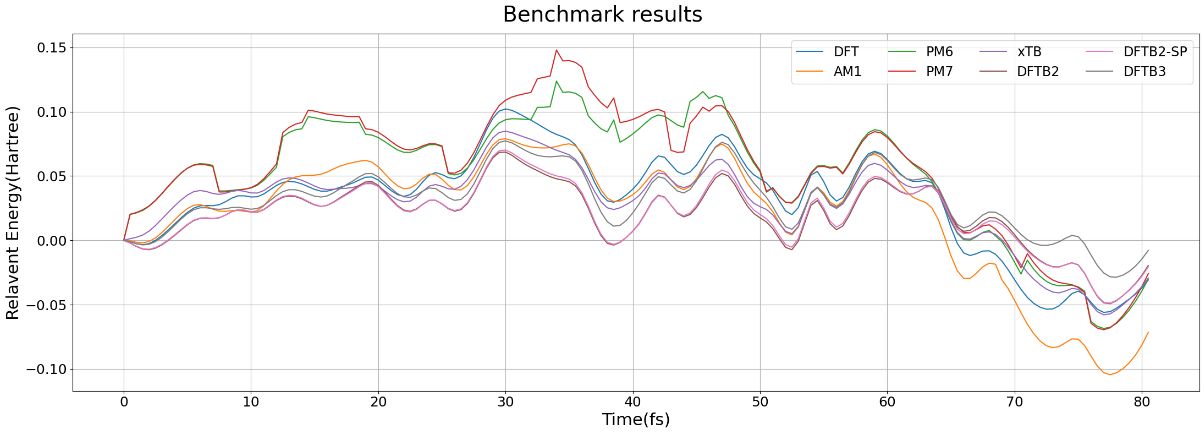

Figure S19: Carbon-6,Trajectory-10.

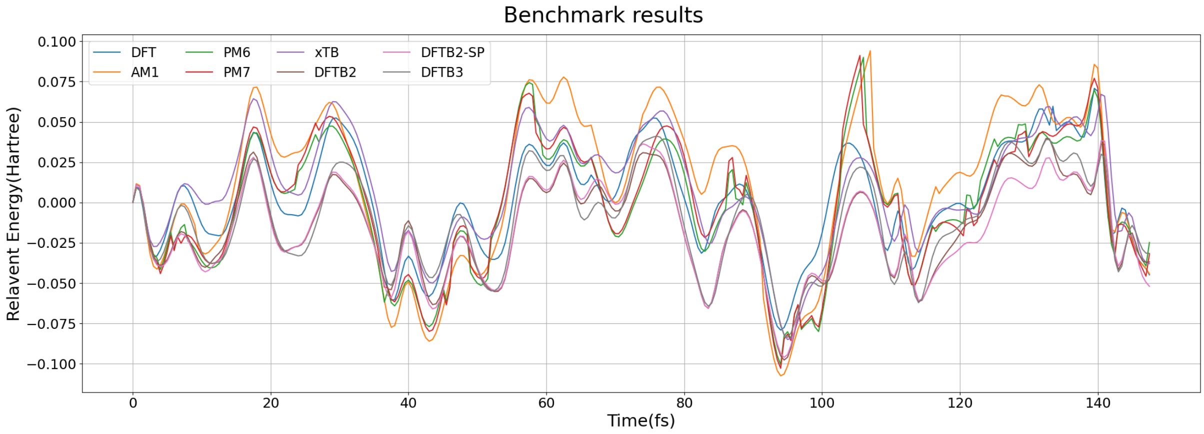

Figure S20: Carbon-6,Trajectory-11.

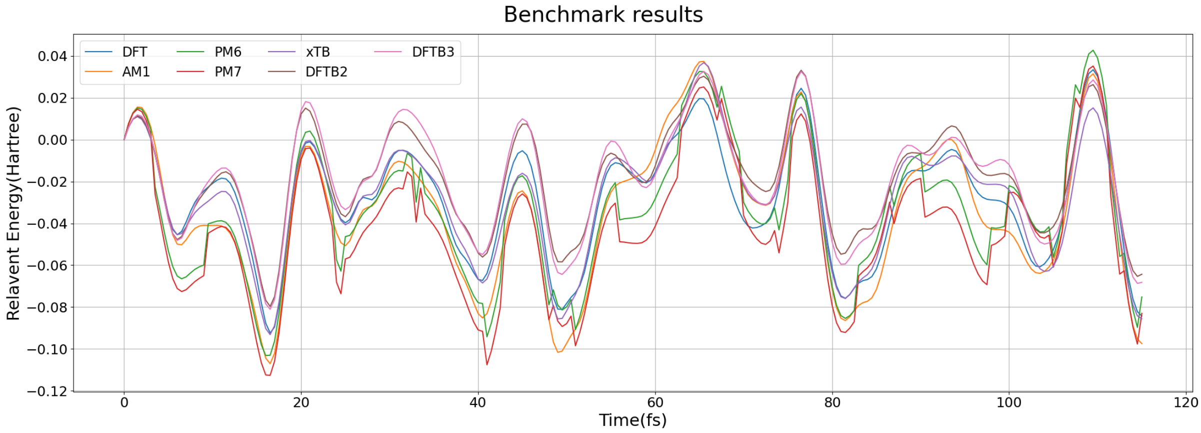

Figure S21: Carbon-6,Trajectory-12.

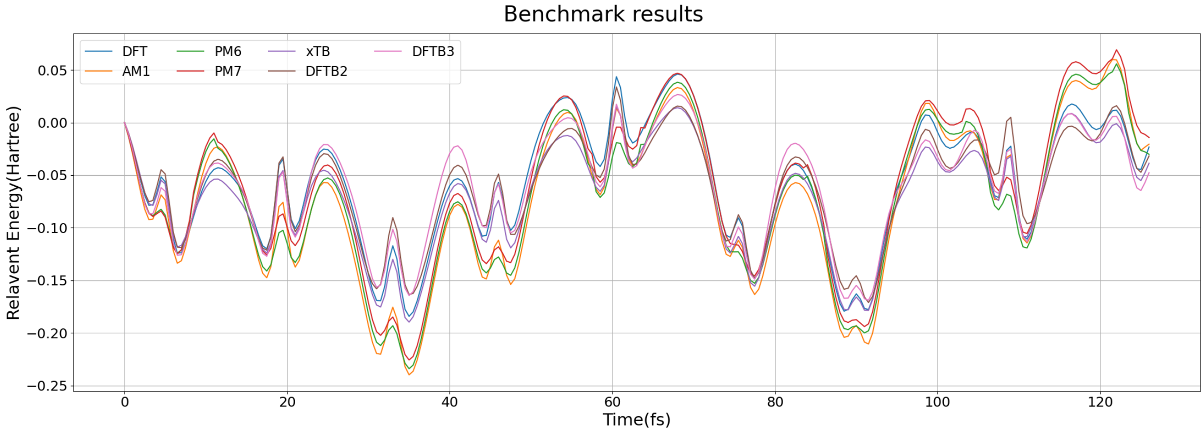

Figure S22: Carbon-6,Trajectory-13.

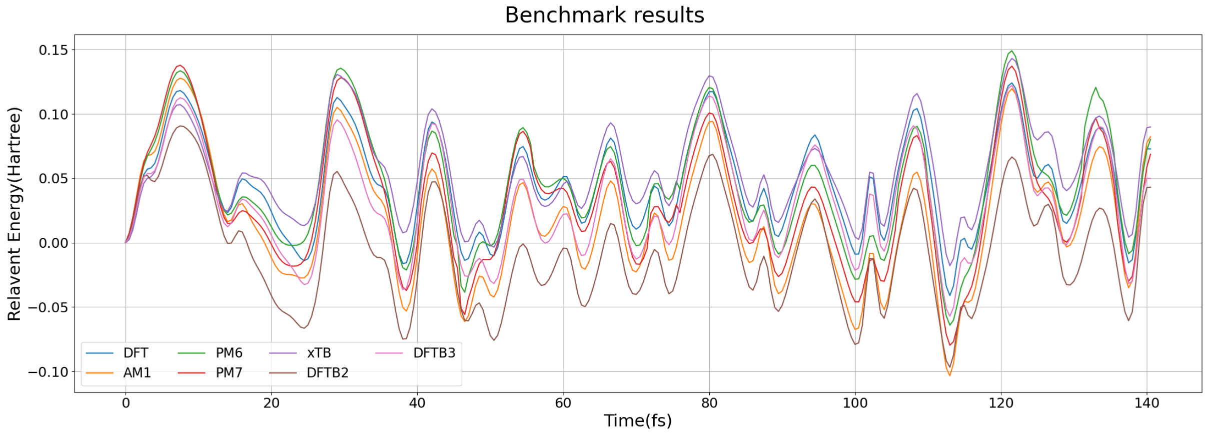

Figure S23: Carbon-6,Trajectory-14.

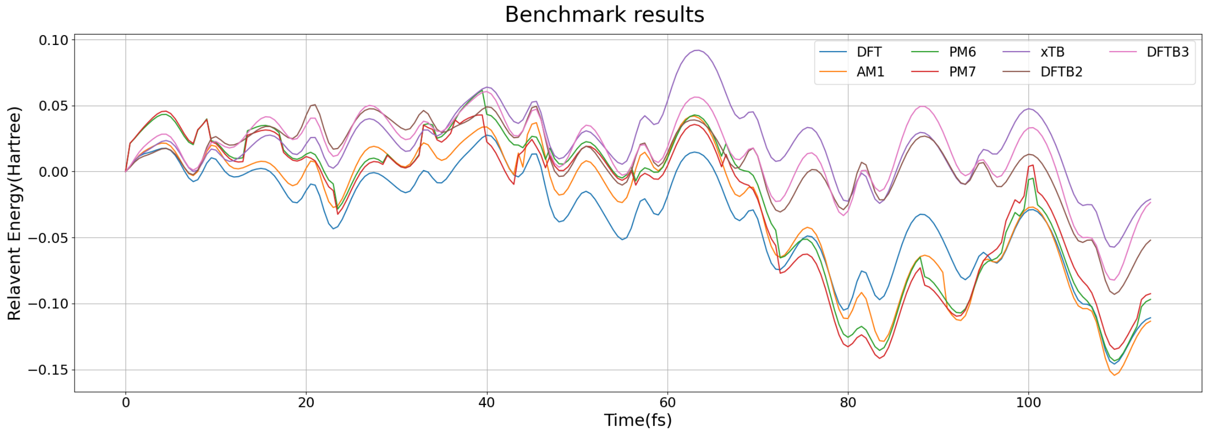

Figure S24: Carbon-6,Trajectory-15.

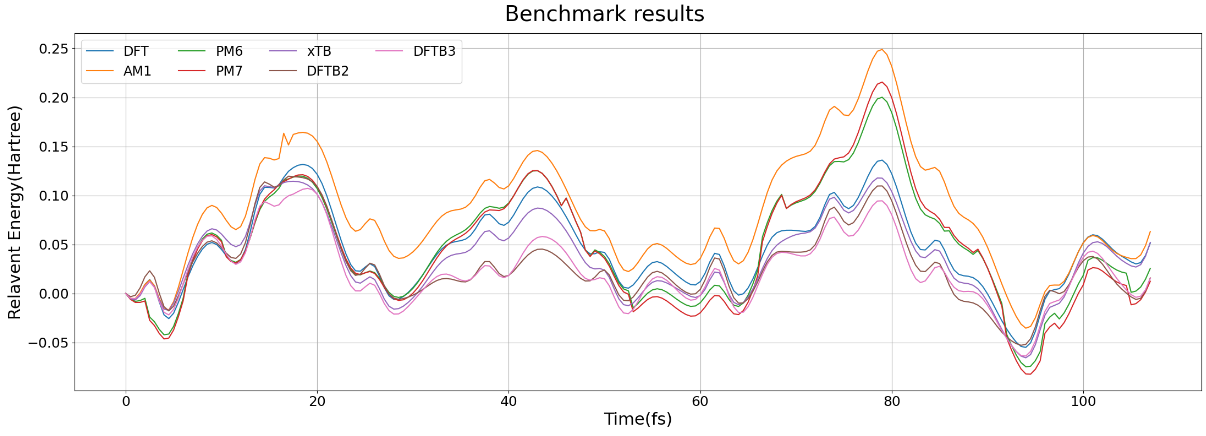

Figure S25: Carbon-6,Trajectory-16.

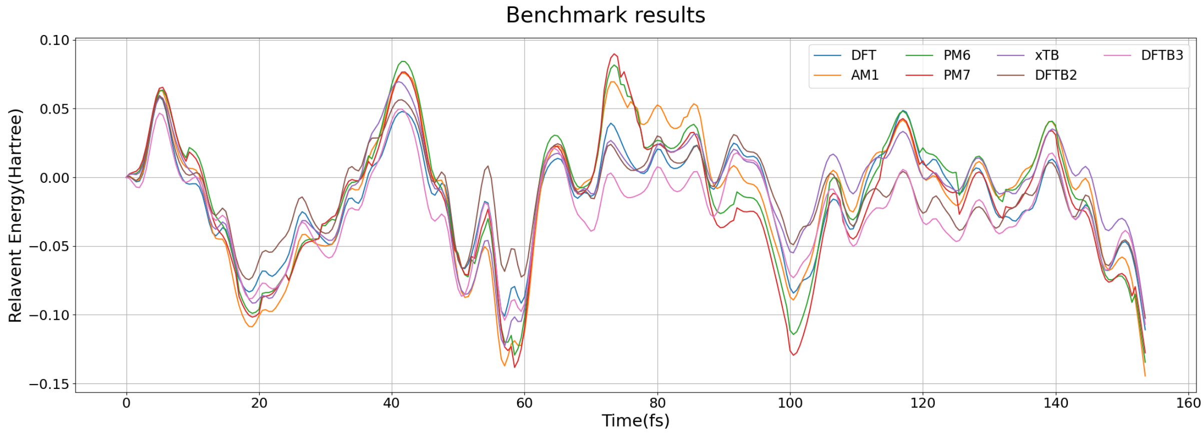

Figure S26: Carbon-6,Trajectory-17.

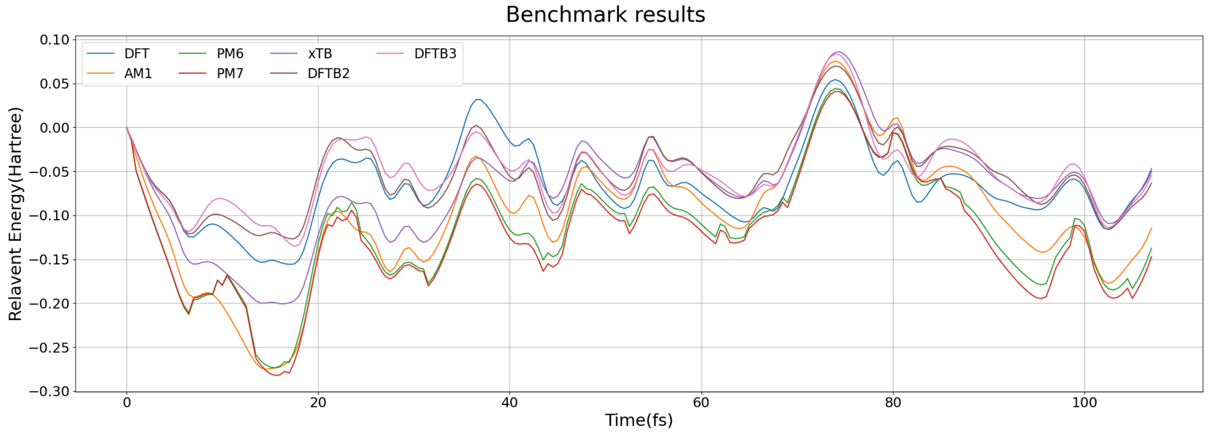

Figure S27: Carbon-6,Trajectory-18.

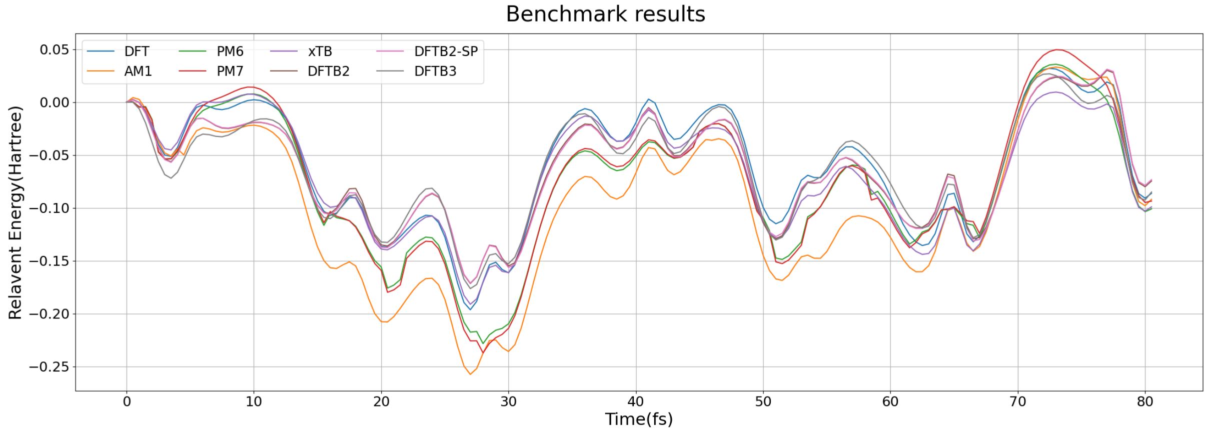

Figure S28: Carbon-7,Trajectory-1.

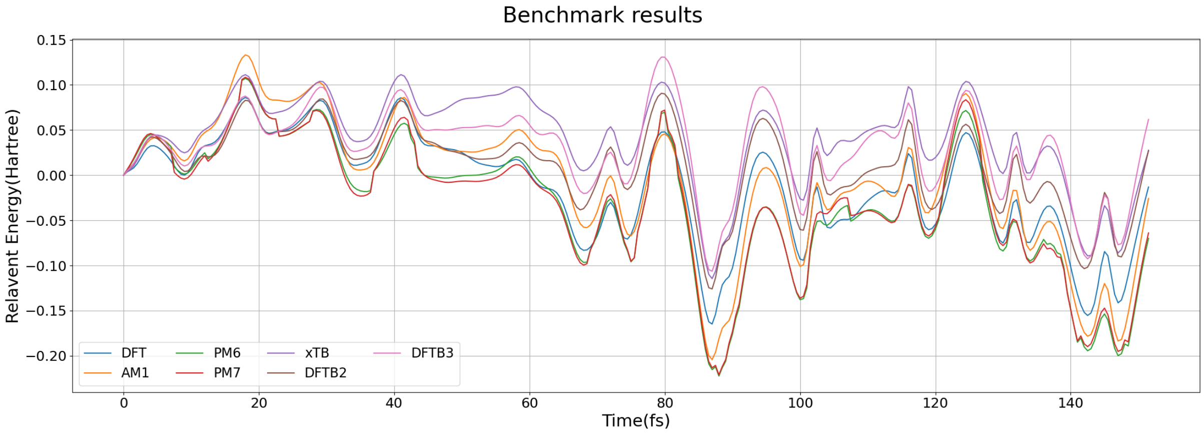

Figure S29: Carbon-7,Trajectory-2.

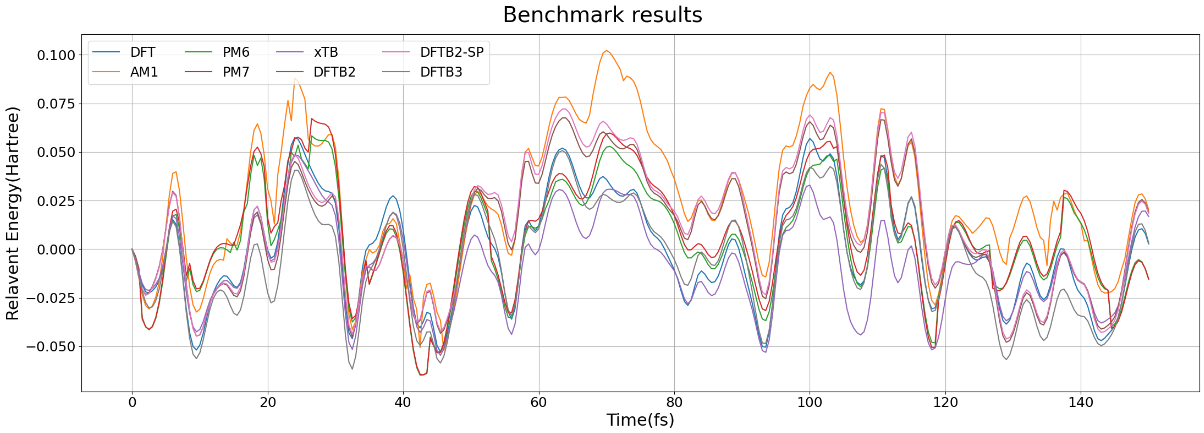

Figure S30: Carbon-7,Trajectory-3.

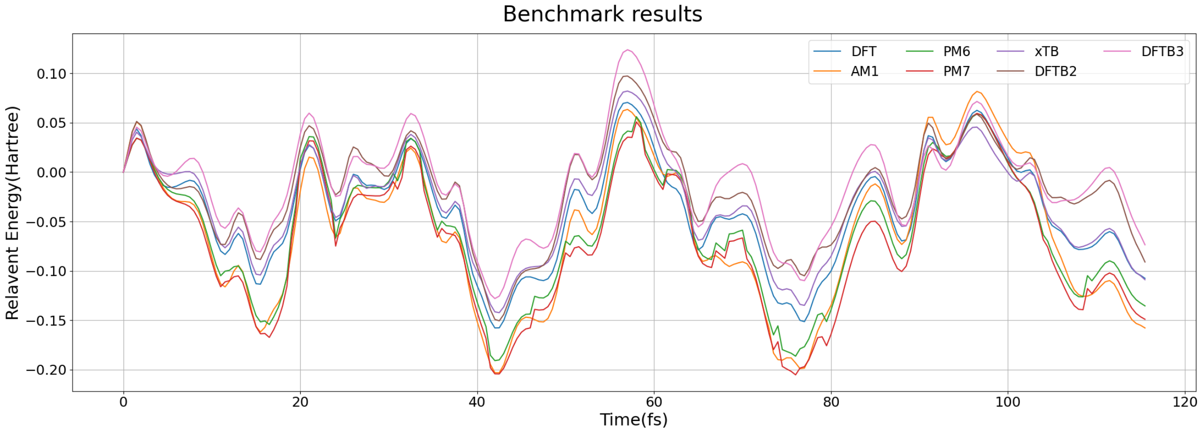

Figure S31: Carbon-7,Trajectory-4.

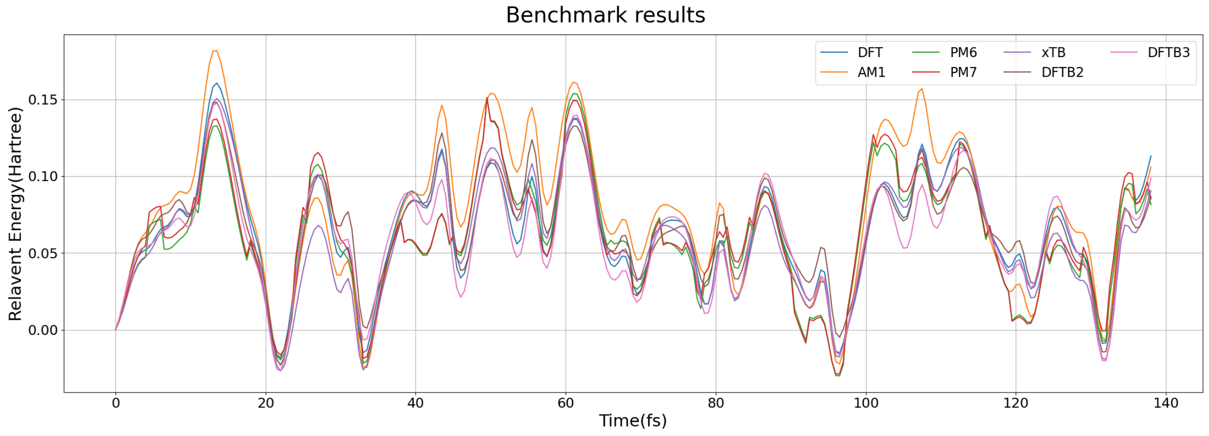

Figure S32: Carbon-7,Trajectory-5.

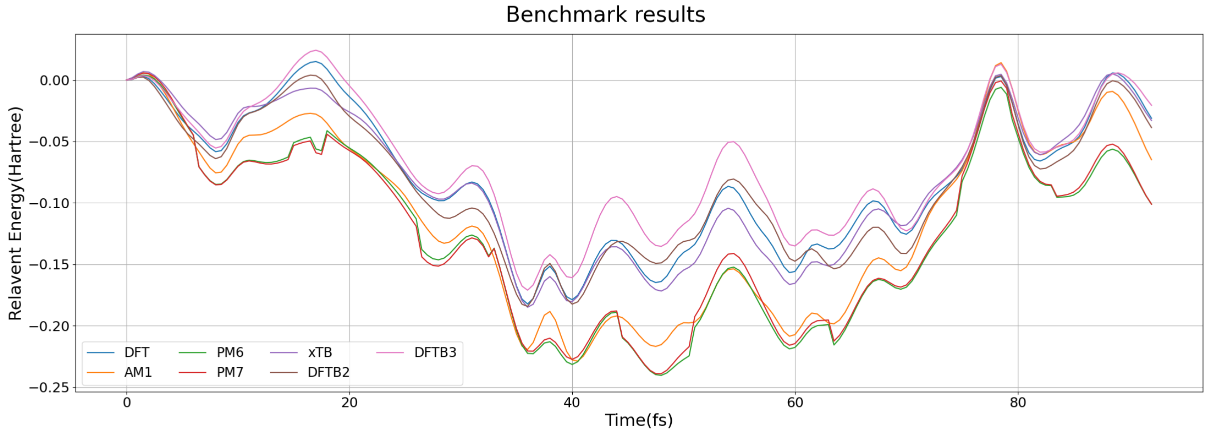

Figure S33: Carbon-7,Trajectory-6.

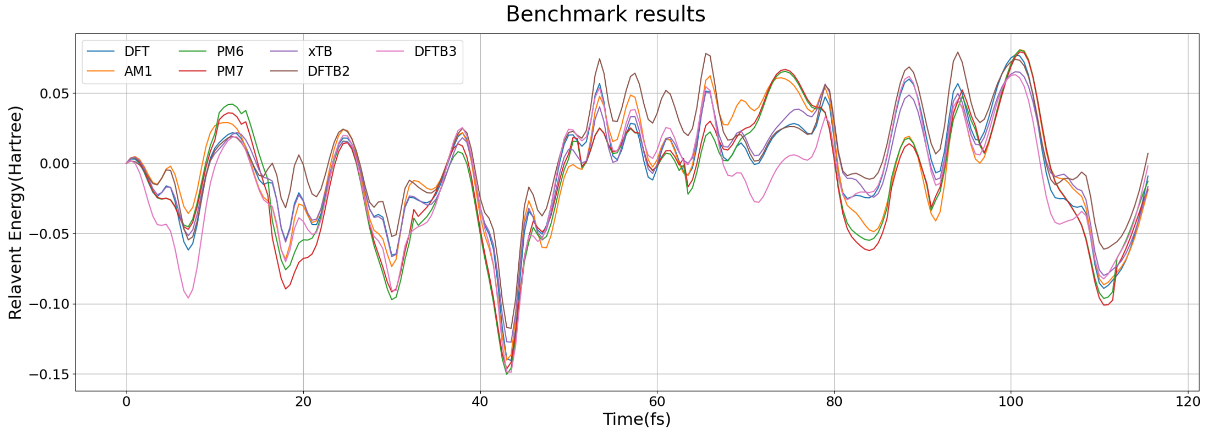

Figure S34: Carbon-7,Trajectory-7.

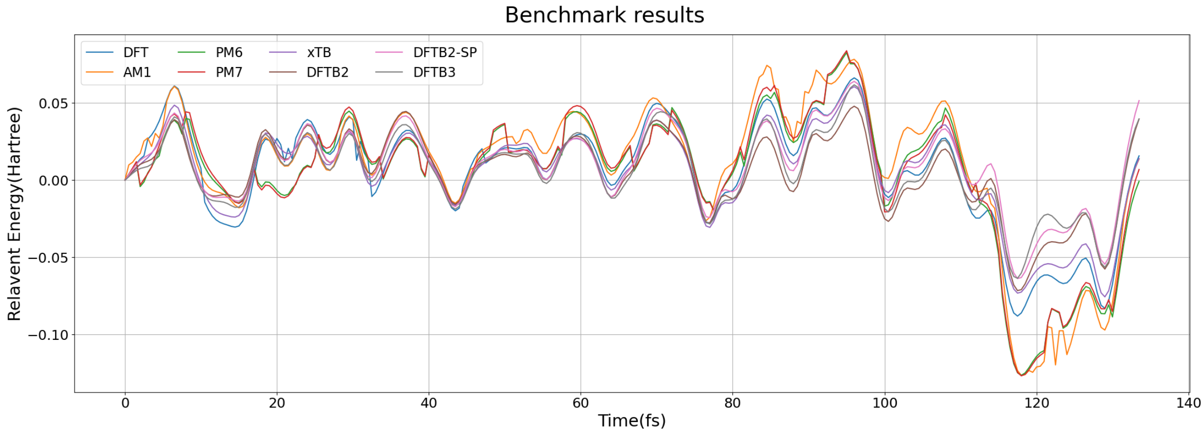

Figure S35: Carbon-7,Trajectory-8.

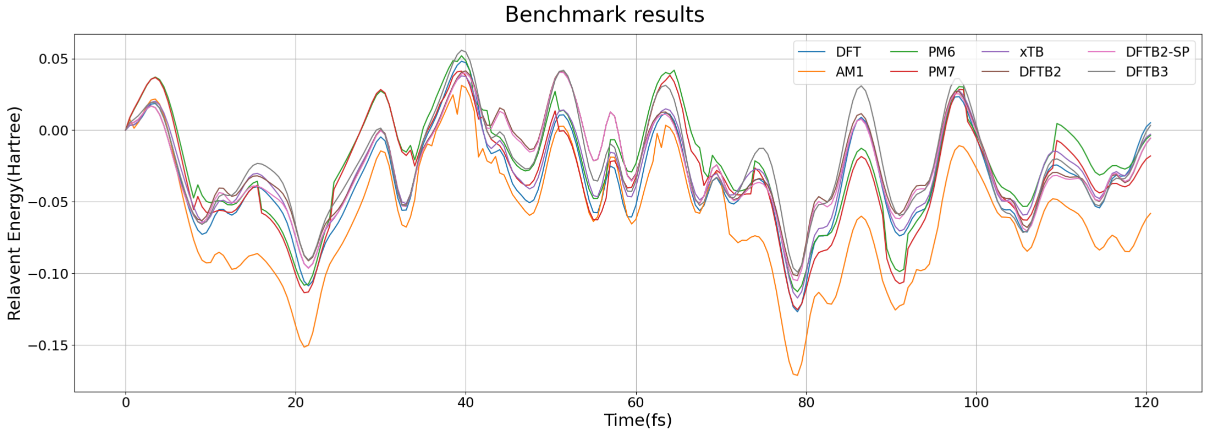

Figure S36: Carbon-7,Trajectory-9.

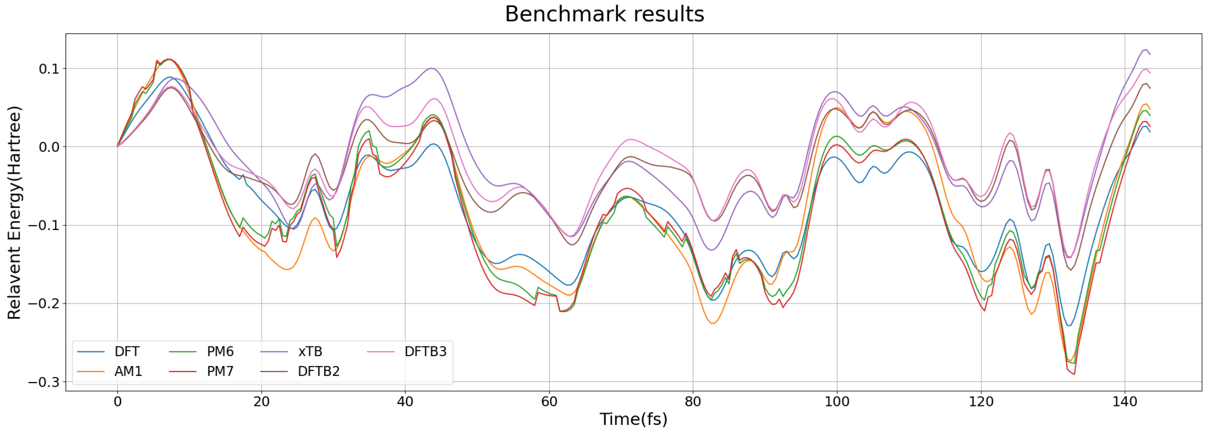

Figure S37: Carbon-8,Trajectory-1.

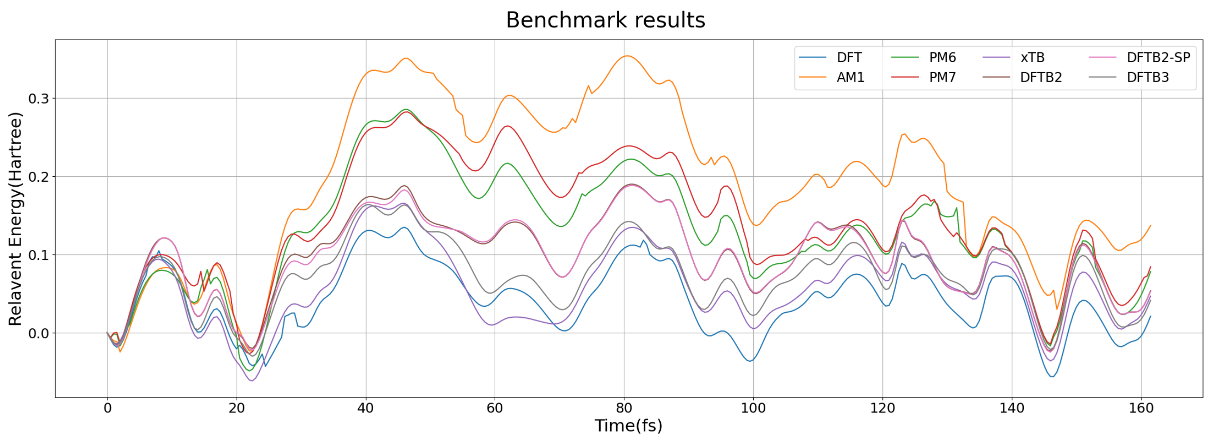

Figure S38: Carbon-8,Trajectory-2.

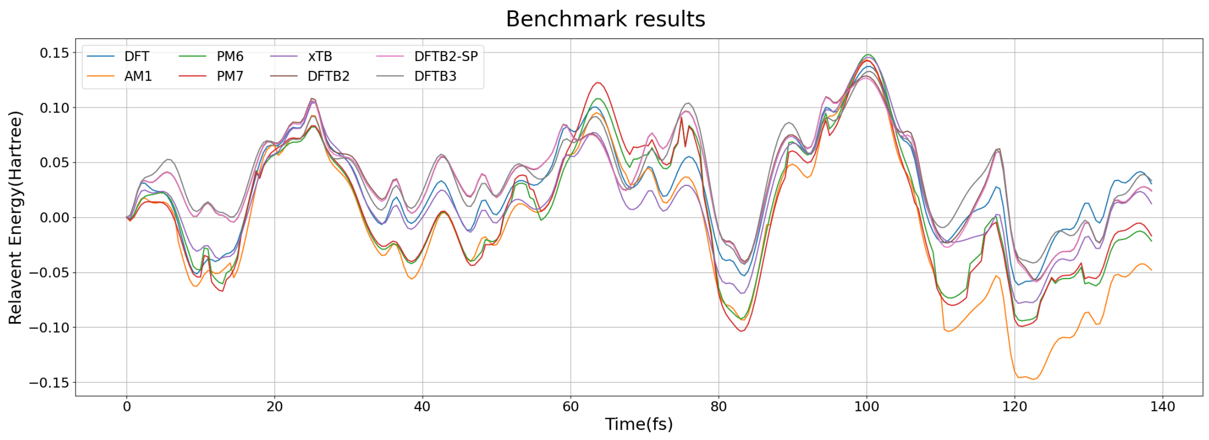

Figure S39: Carbon-8,Trajectory-3.

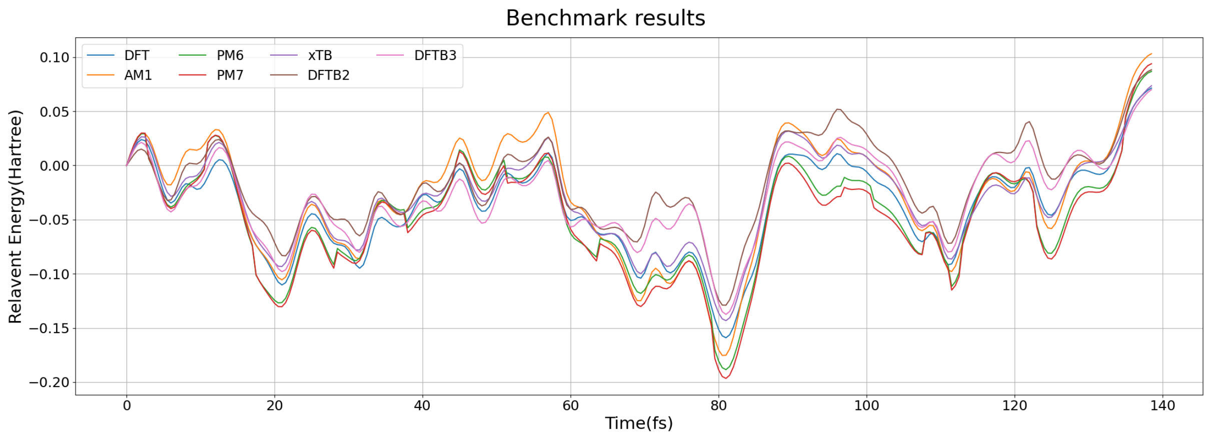

Figure S40: Carbon-8,Trajectory-4.

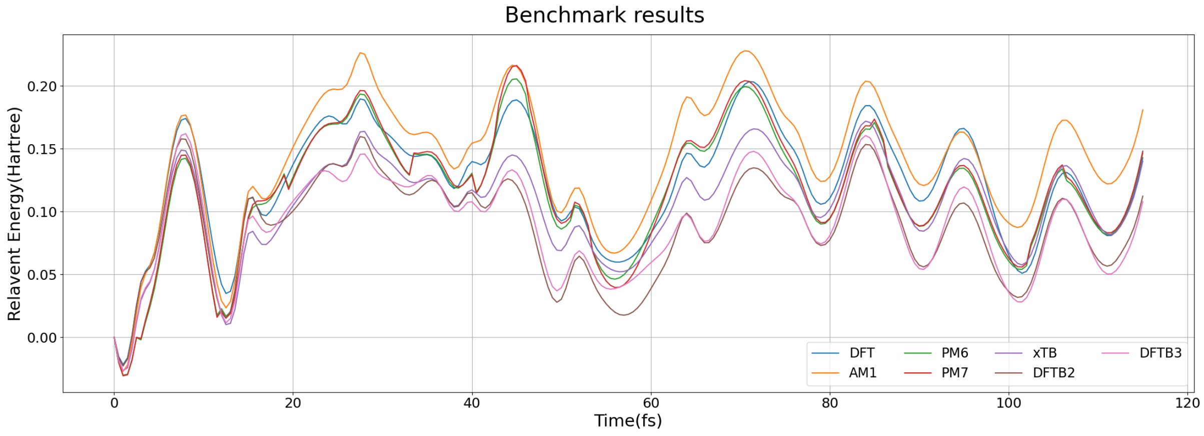

Figure S41: Carbon-8,Trajectory-5.

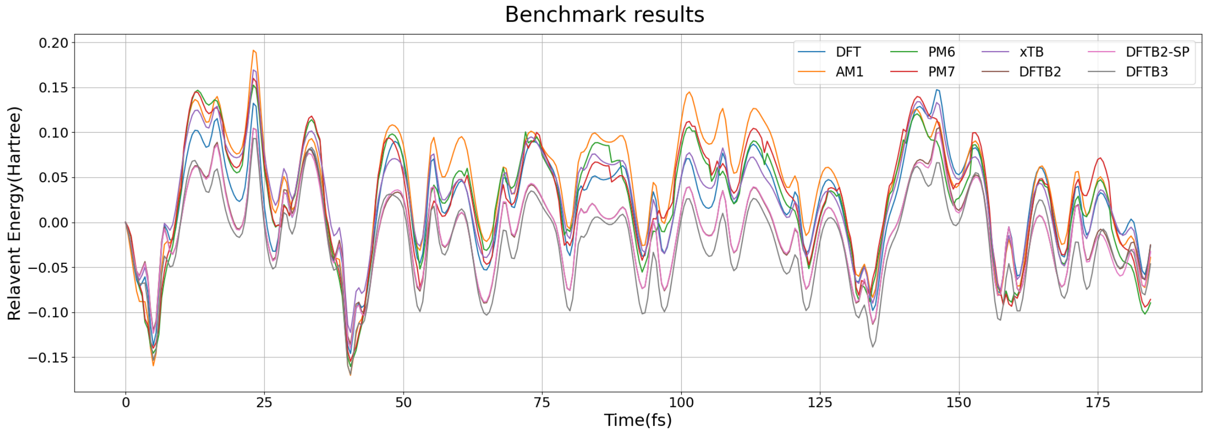

Figure S42: Carbon-8,Trajectory-6.

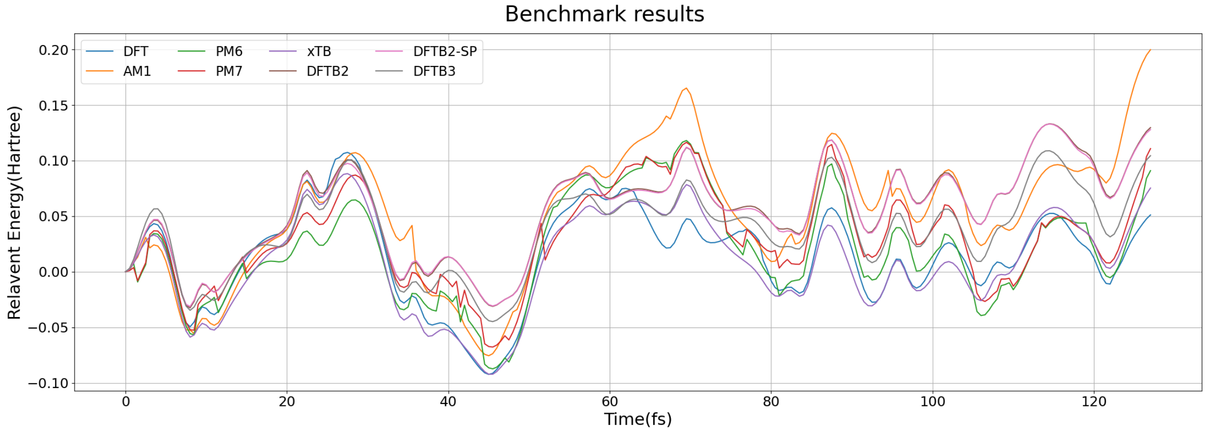

Figure S43: Carbon-8,Trajectory-7.

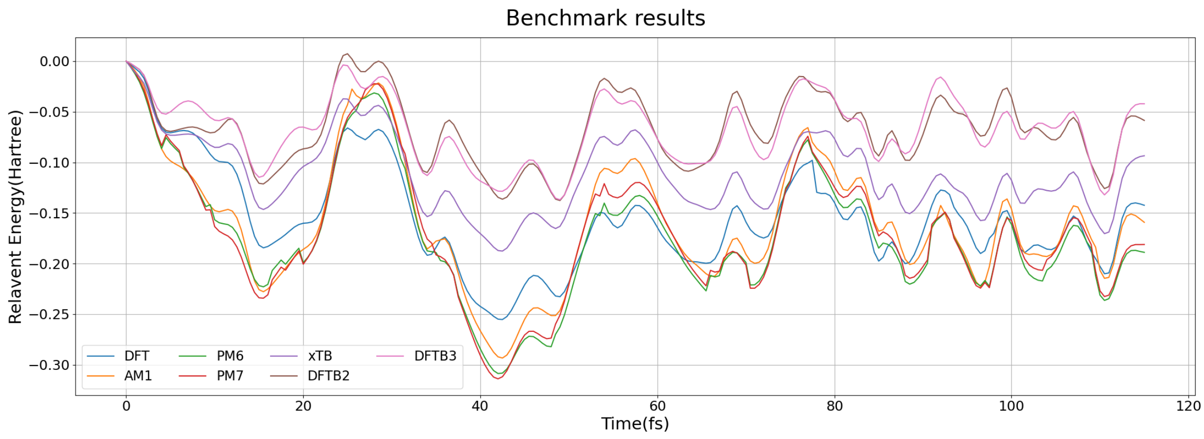

Figure S44: Carbon-10,Trajectory-1.

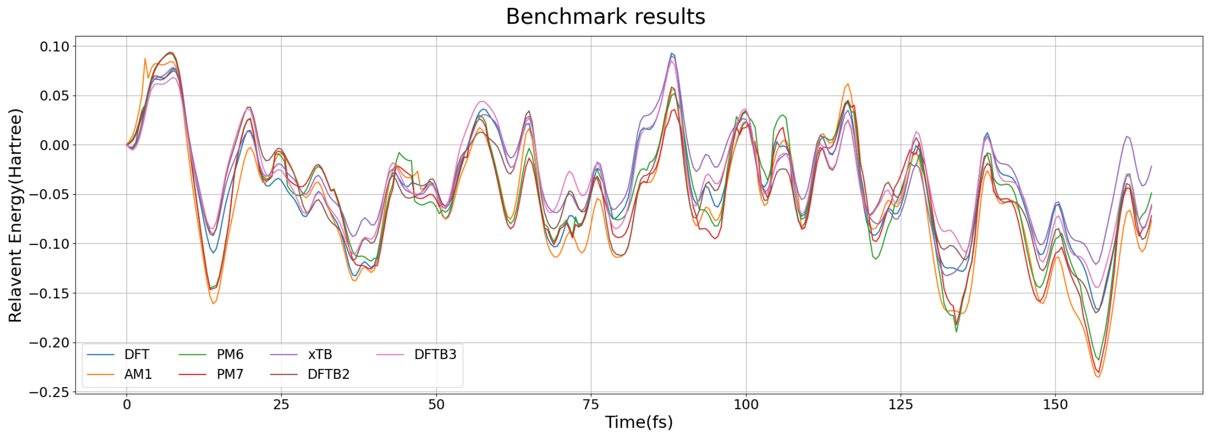

Figure S45: Carbon-10,Trajectory-2.

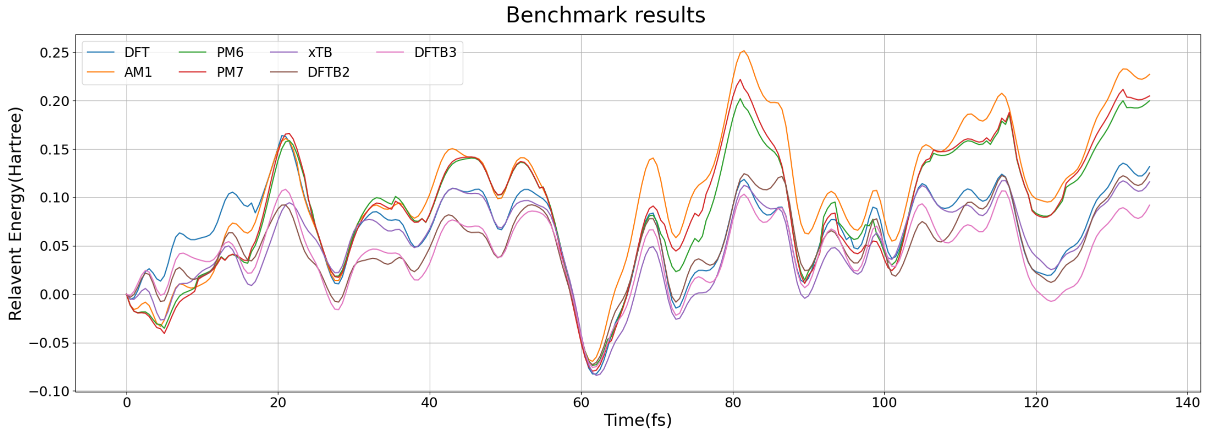

Figure S46: Carbon-10,Trajectory-3.

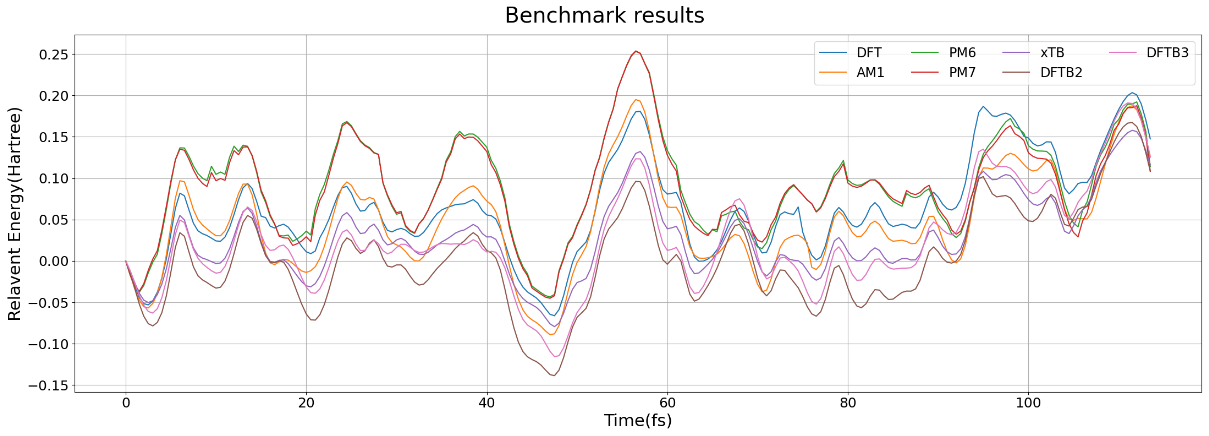

Figure S47: Carbon-10,Trajectory-4.

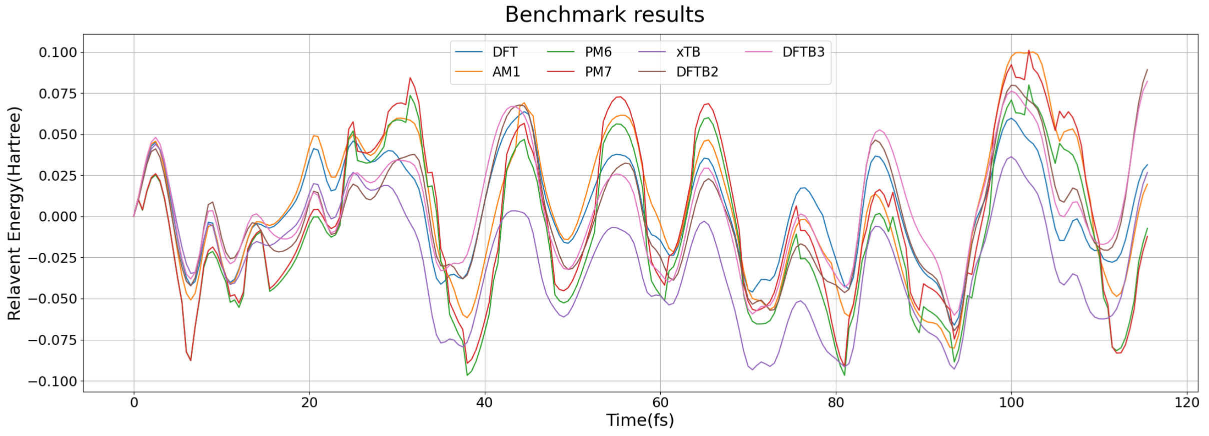

Figure S48: Carbon-10,Trajectory-5.

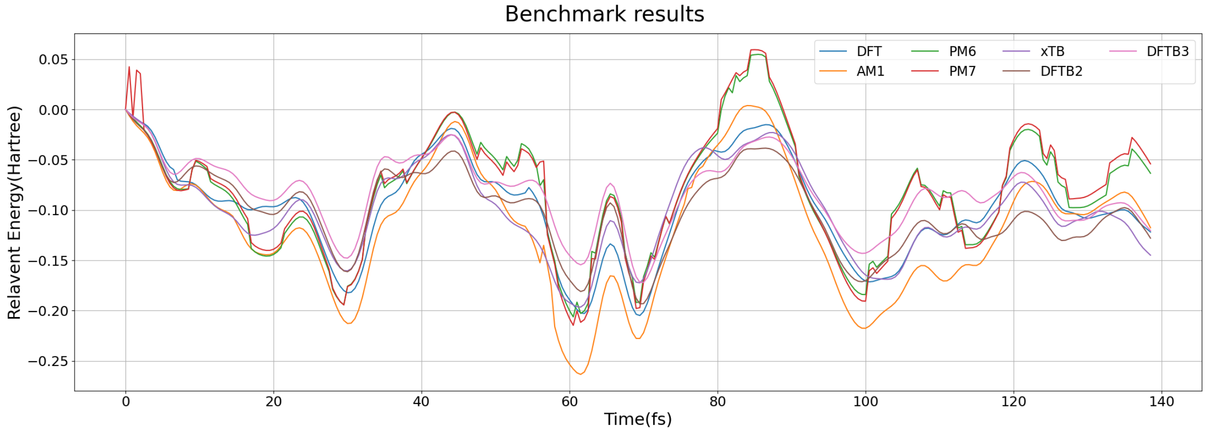

Figure S49: Carbon-10,Trajectory-6.

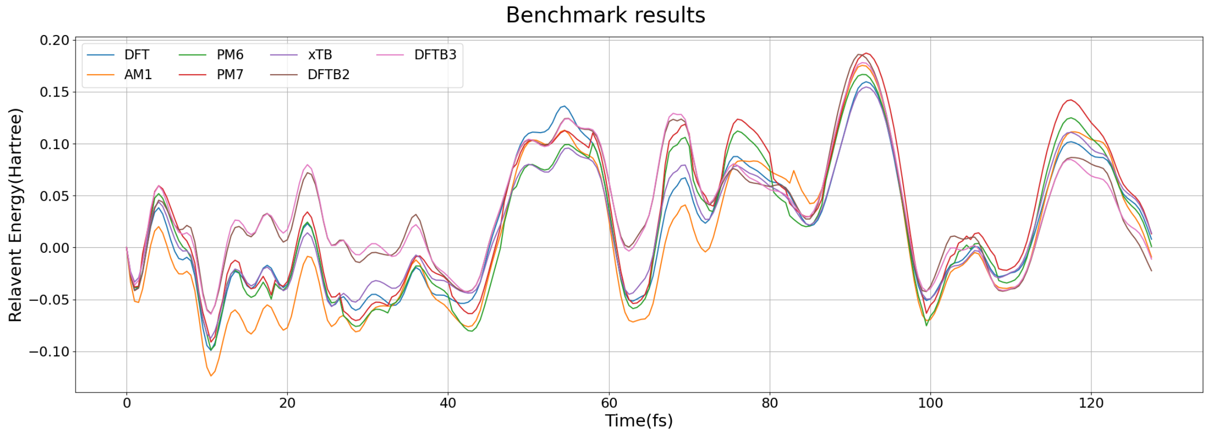

Figure S50: Carbon-10,Oxygen-1,Trajectory-7.

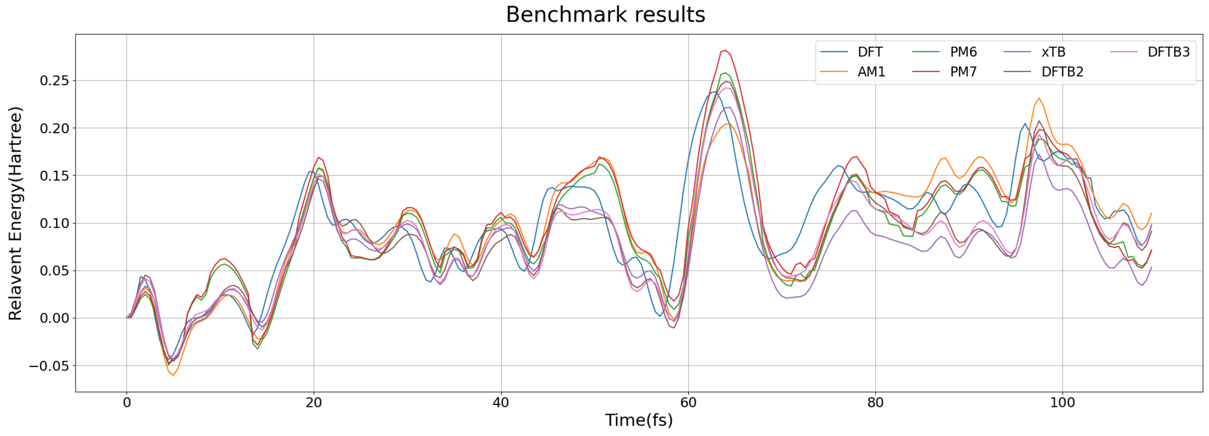

Figure S51: Carbon-11,Trajectory-1.

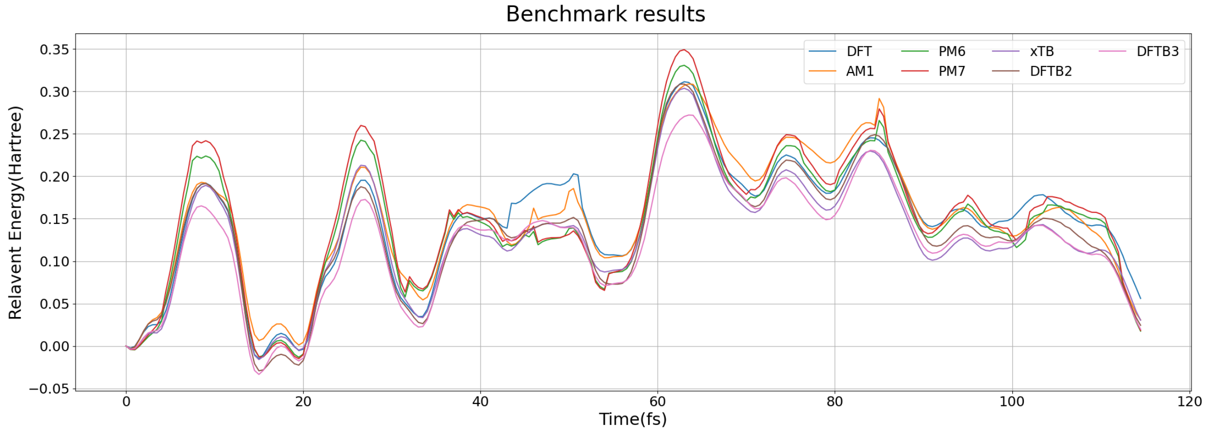

Figure S52: Carbon-11,Trajectory-2.

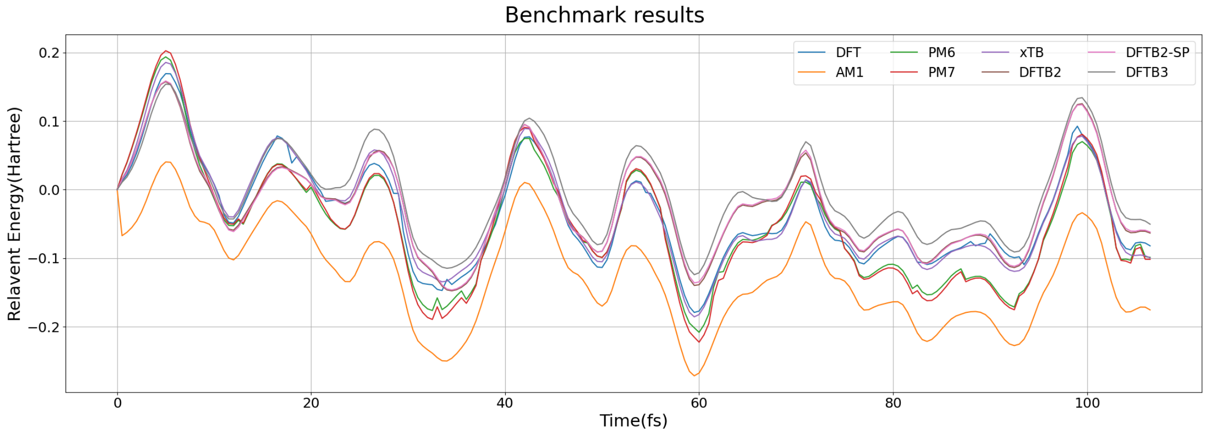

Figure S53: Carbon-11,Trajectory-3.

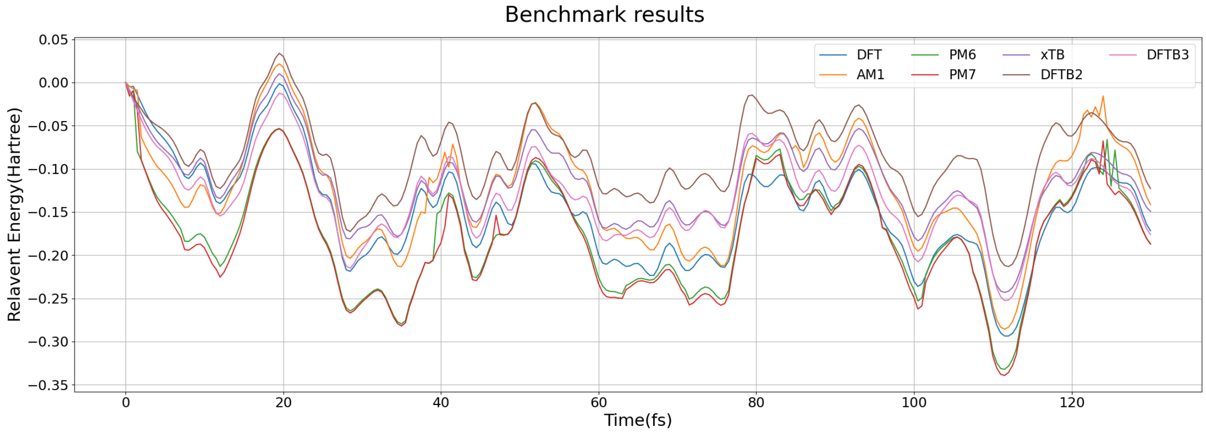

Figure S54: Carbon-11,Trajectory-4.

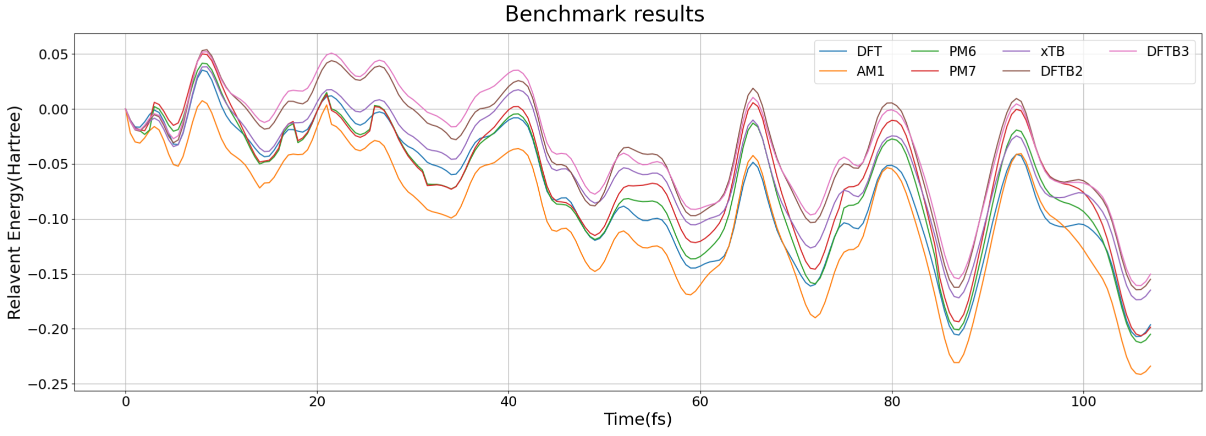

Figure S55: Carbon-11,Trajectory-5.

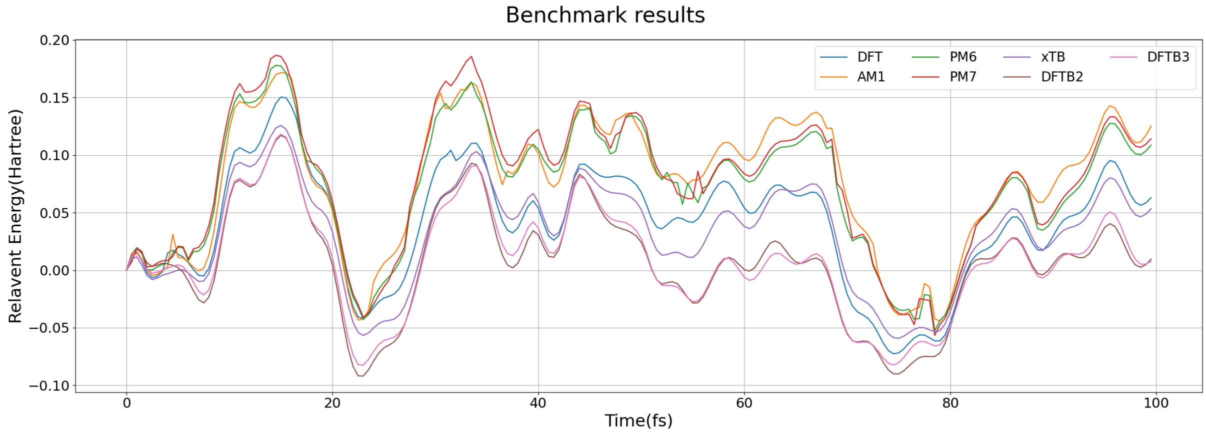

Figure S56: Carbon-12,Trajectory-1.

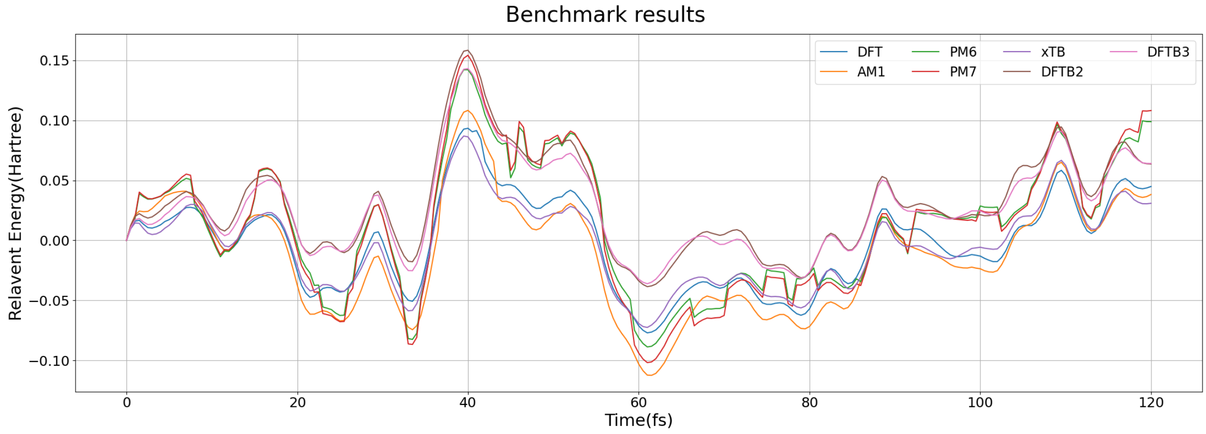

Figure S57: Carbon-12,Oxygen-1,Trajectory-2.

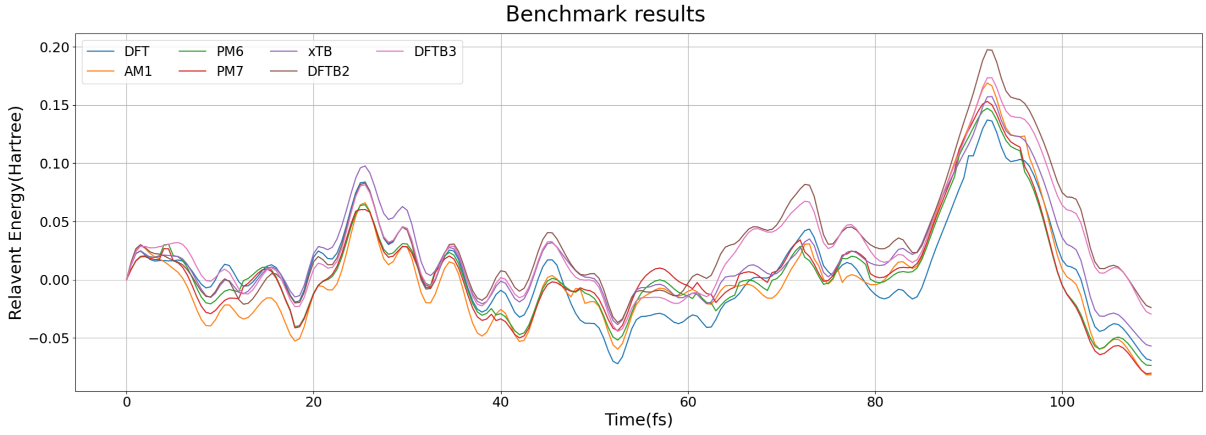

Figure S58: Carbon-12,Trajectory-3.

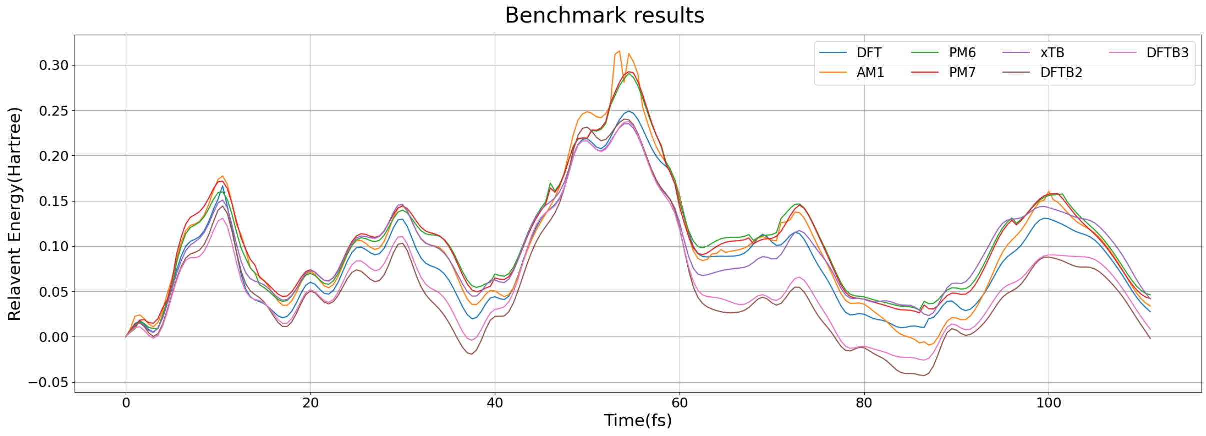

Figure S59: Carbon-12,Trajectory-4.

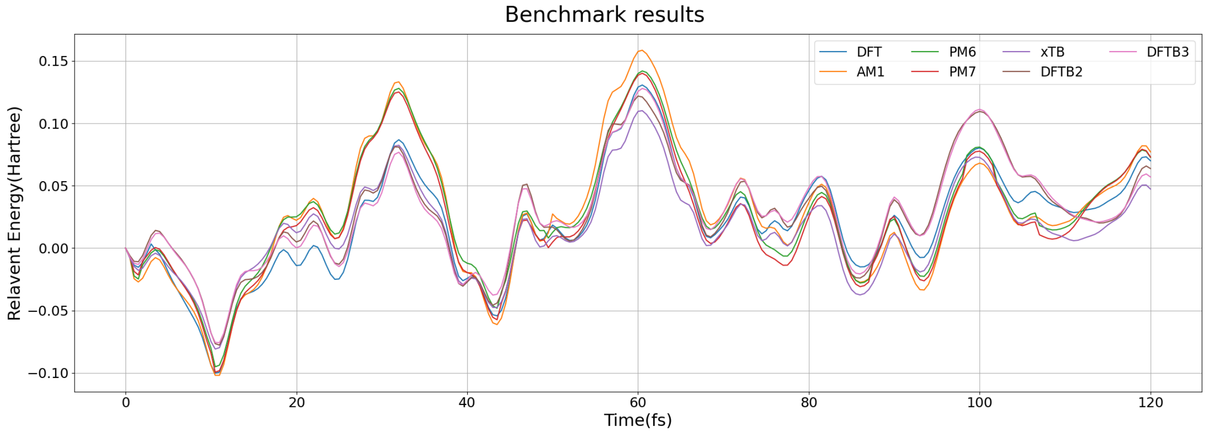

Figure S60: Carbon-12,Trajectory-5.

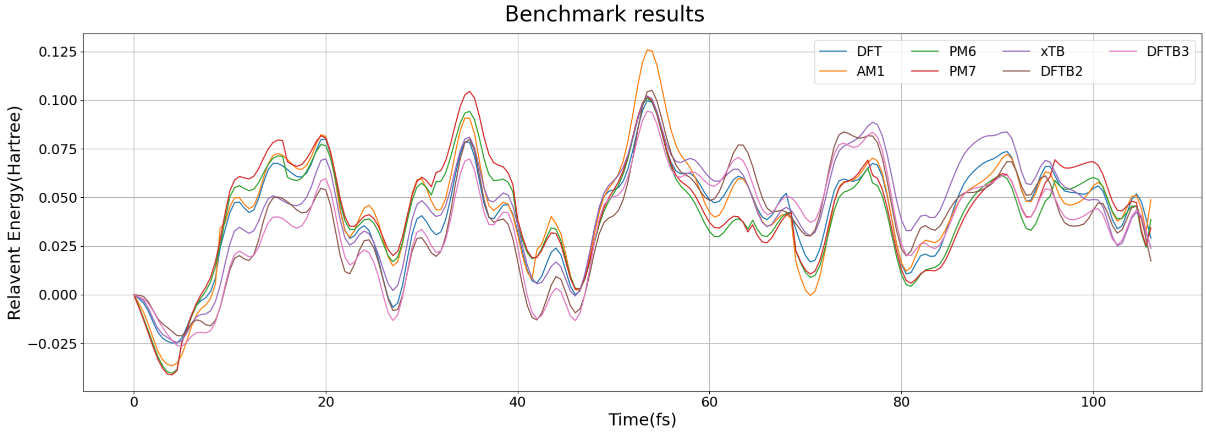

Figure S61: Carbon-12,Trajectory-6.

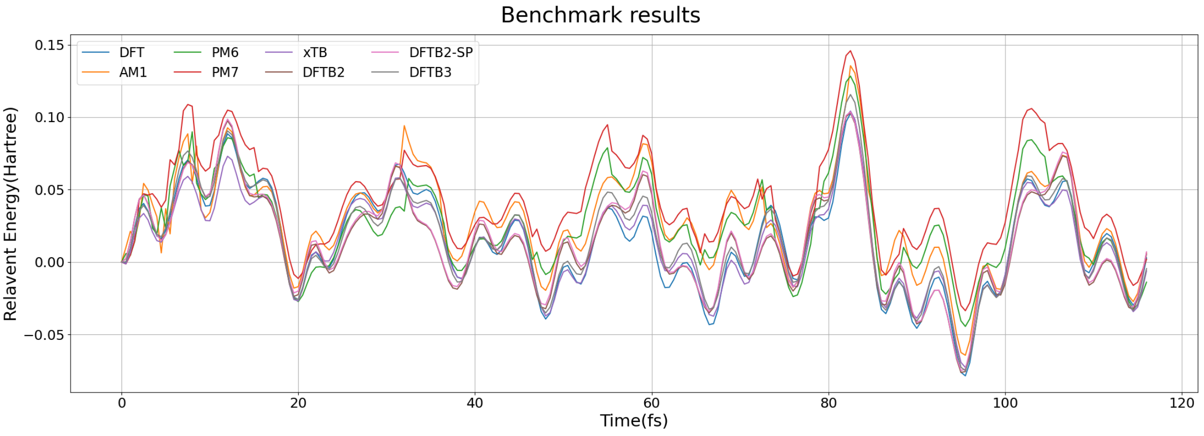

Figure S62: Carbon-12,Trajectory-7.

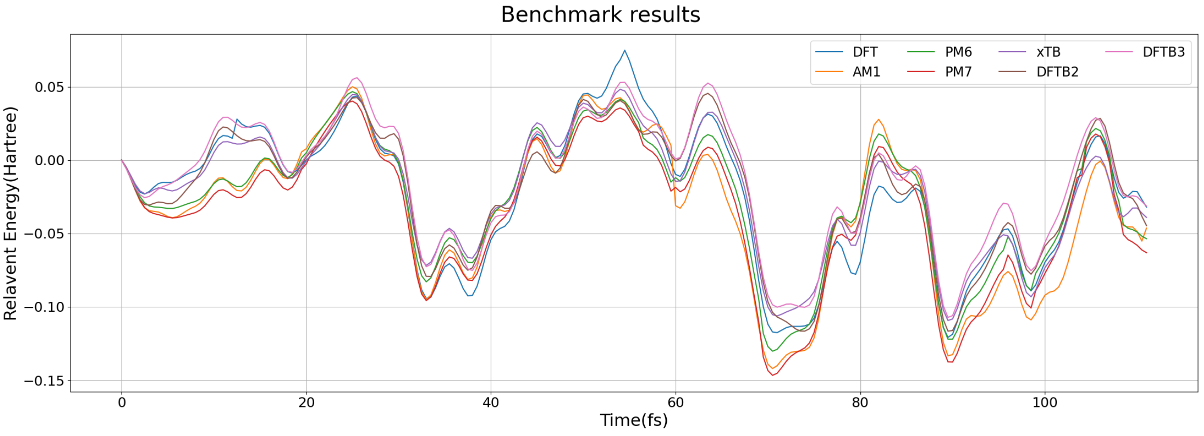

Figure S63: Carbon-14,Trajectory-1.

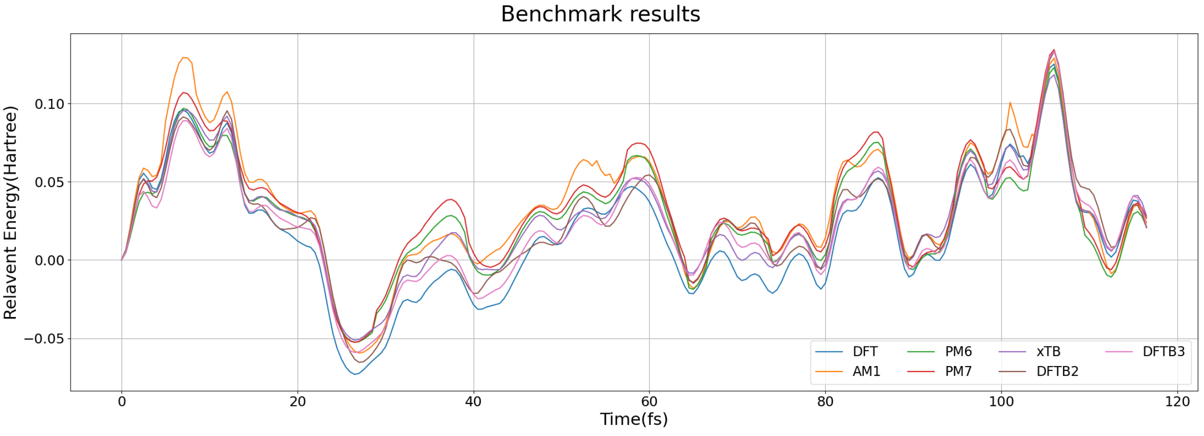

Figure S64: Carbon-15,Trajectory-1.

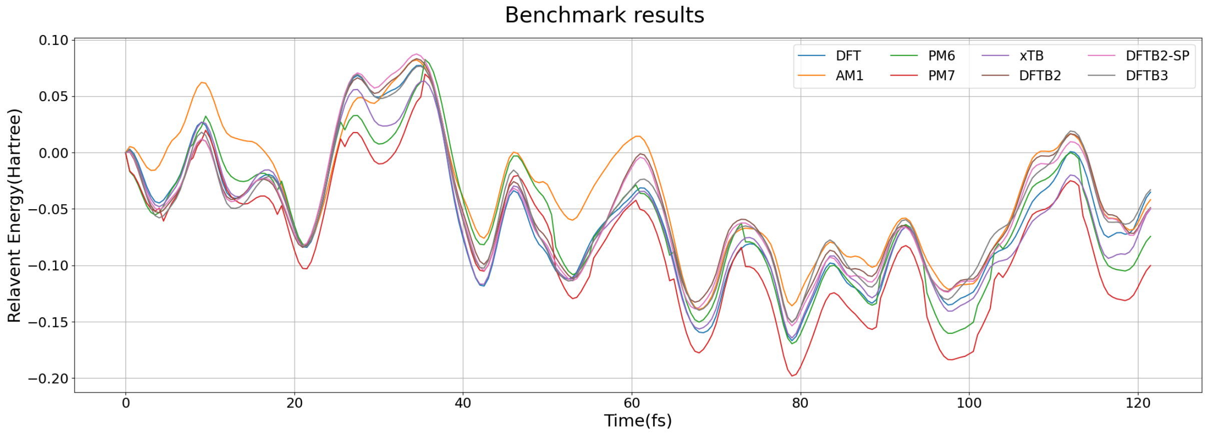

Figure S65: Carbon-16,Trajectory-1.

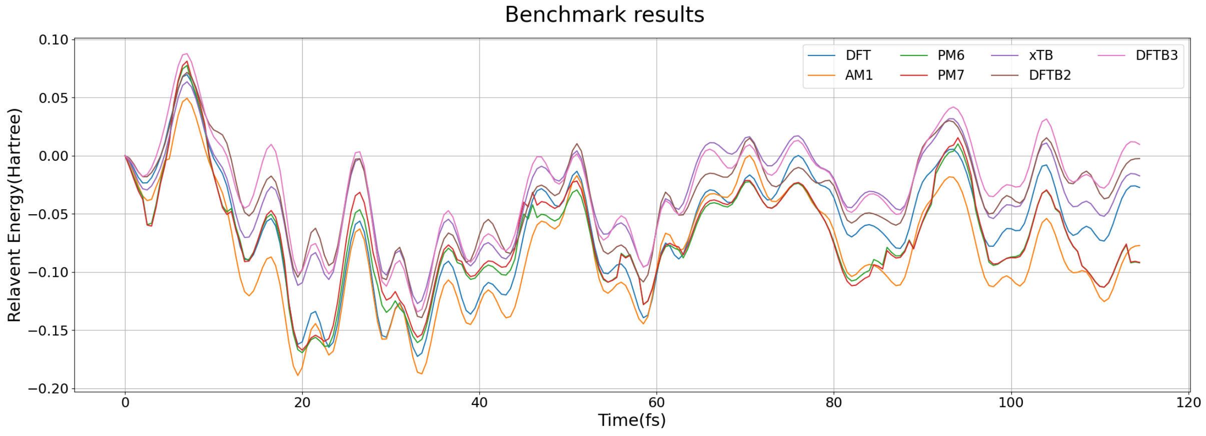

Figure S66: Carbon-16,Trajectory-2.

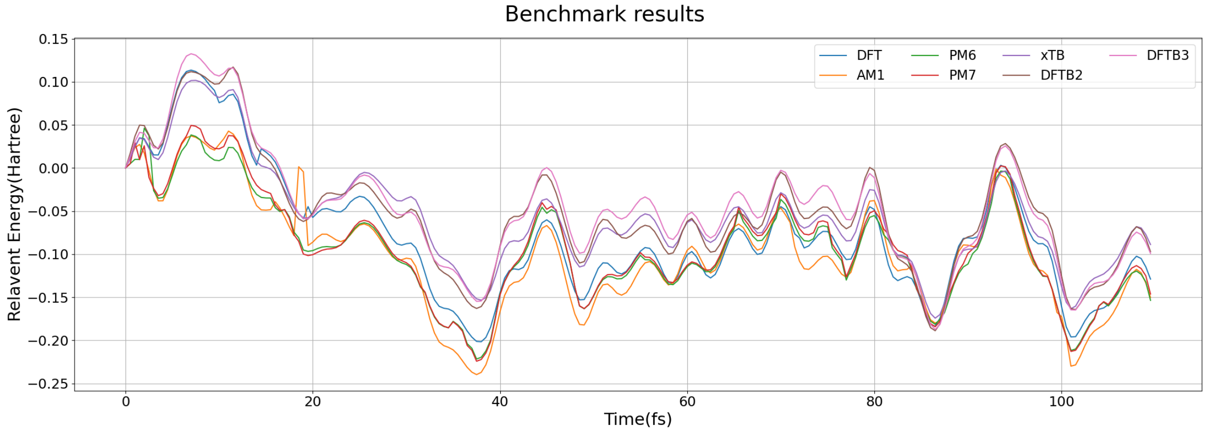

Figure S67: Carbon-16,Trajectory-3.

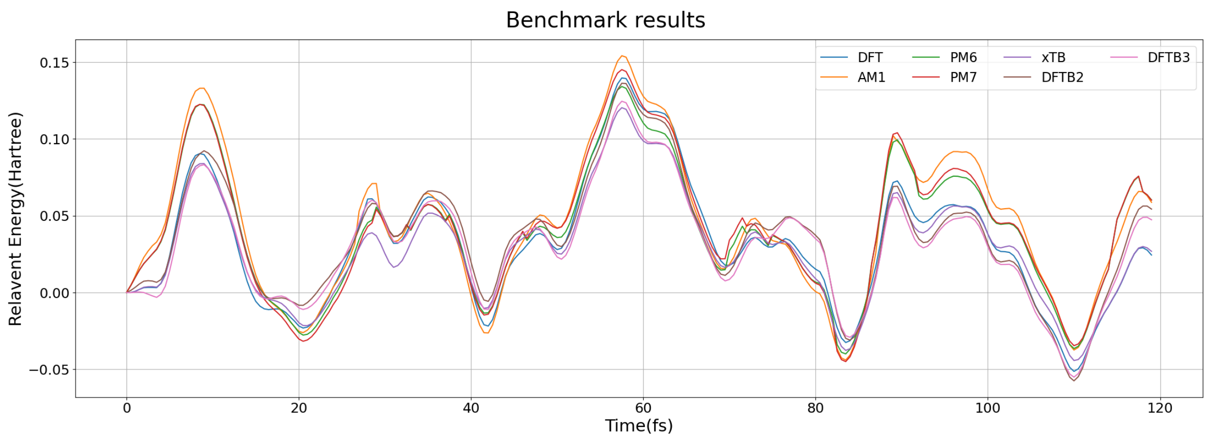

Figure S68: Carbon-16,Trajectory-4.

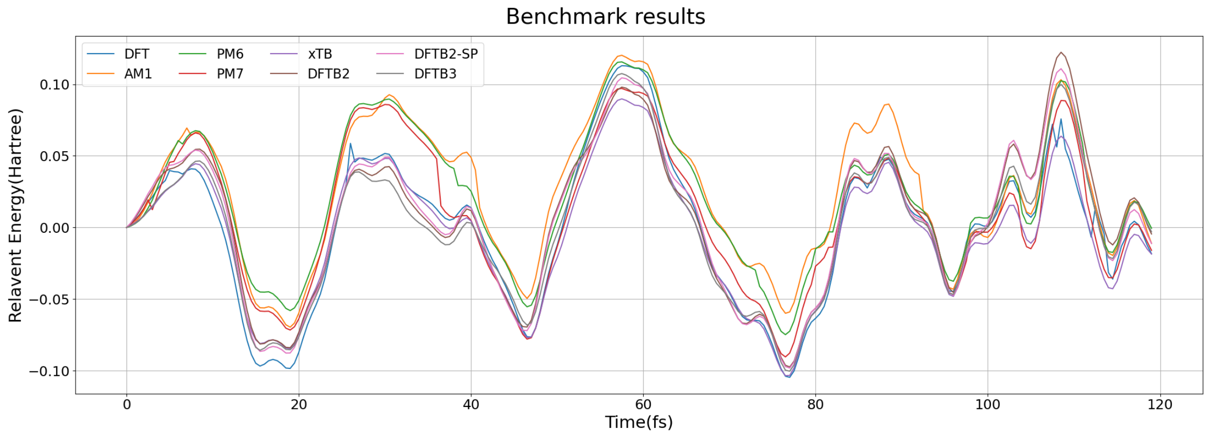

Figure S69: Carbon-16,Trajectory-5.

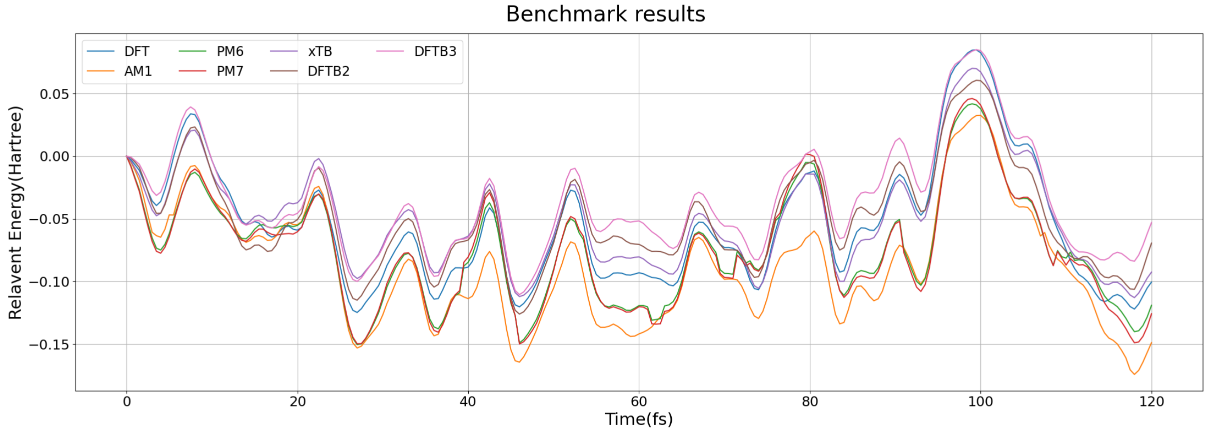

Figure S70: Carbon-17,Trajectory-1.

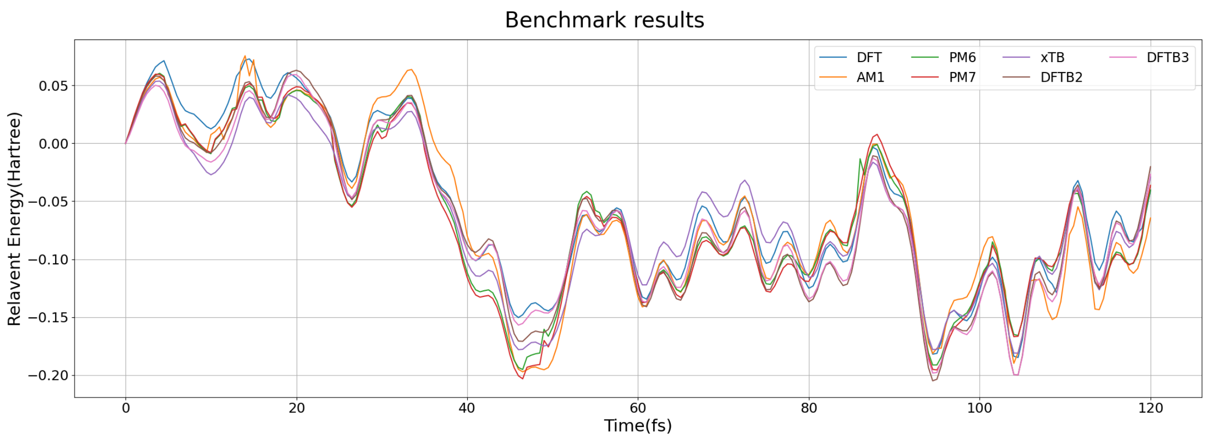

Figure S71: Carbon-17,Trajectory-2.

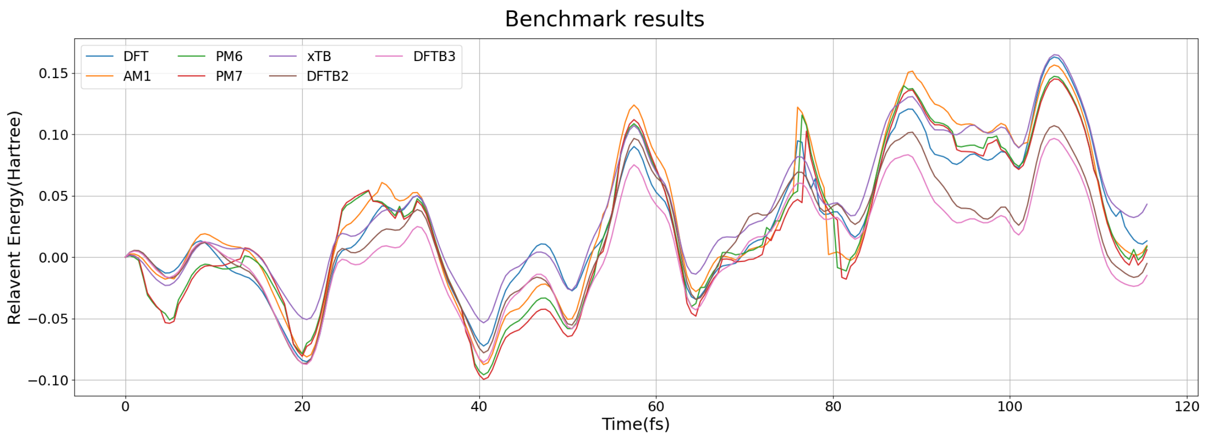

Figure S72: Carbon-17,Trajectory-3.

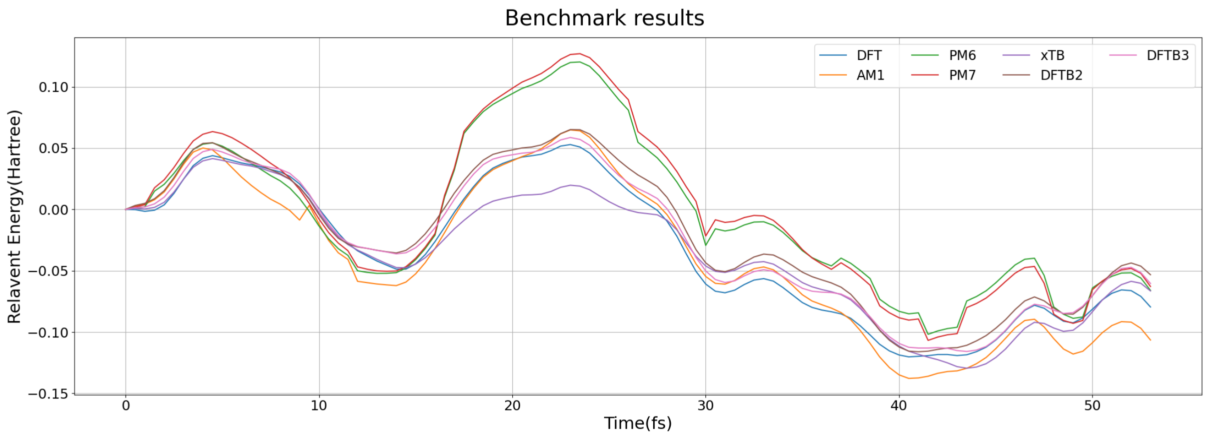

Figure S73: Carbon-17,Oxygen-1,Trajectory-4.

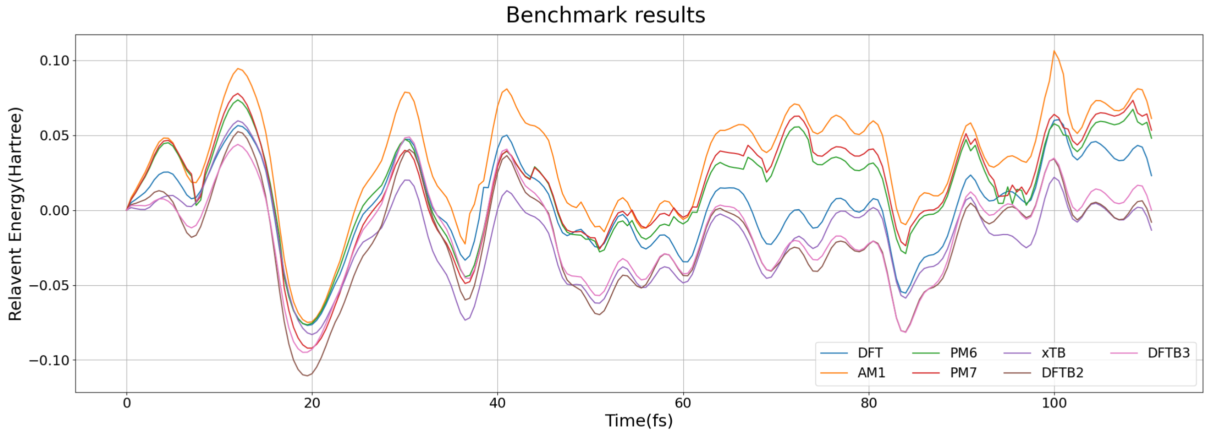

Figure S74: Carbon-17,Trajectory-5.

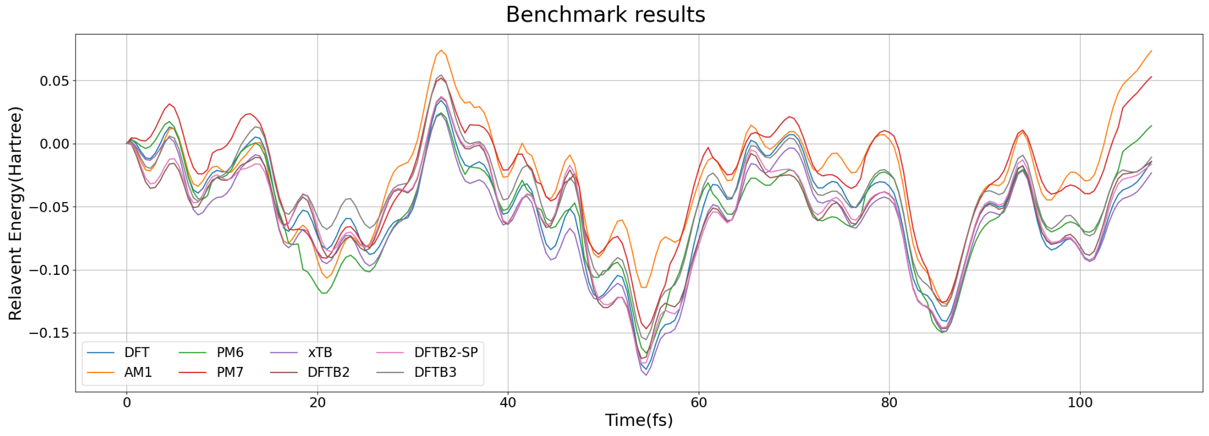

Figure S75: Carbon-17,Trajectory-6.

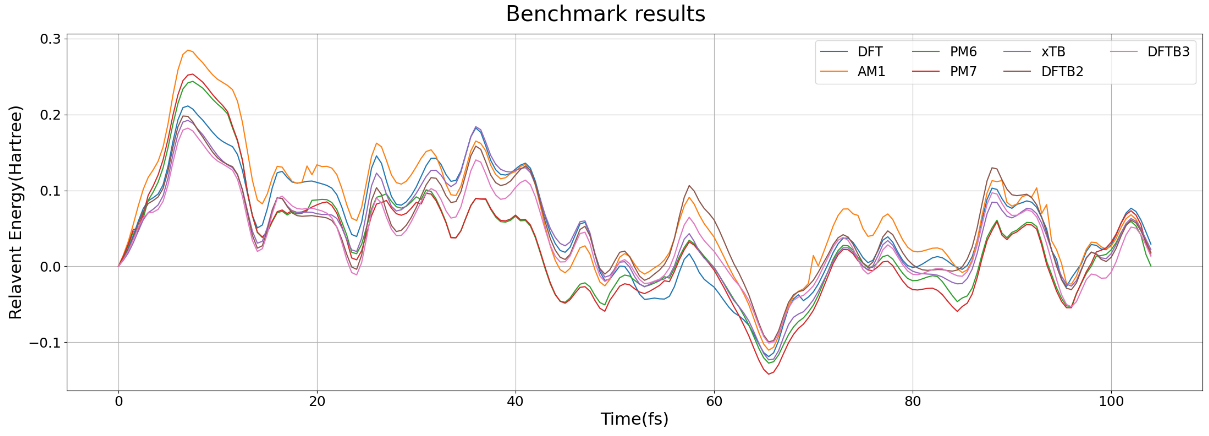

Figure S76: Carbon-19,Trajectory-1.

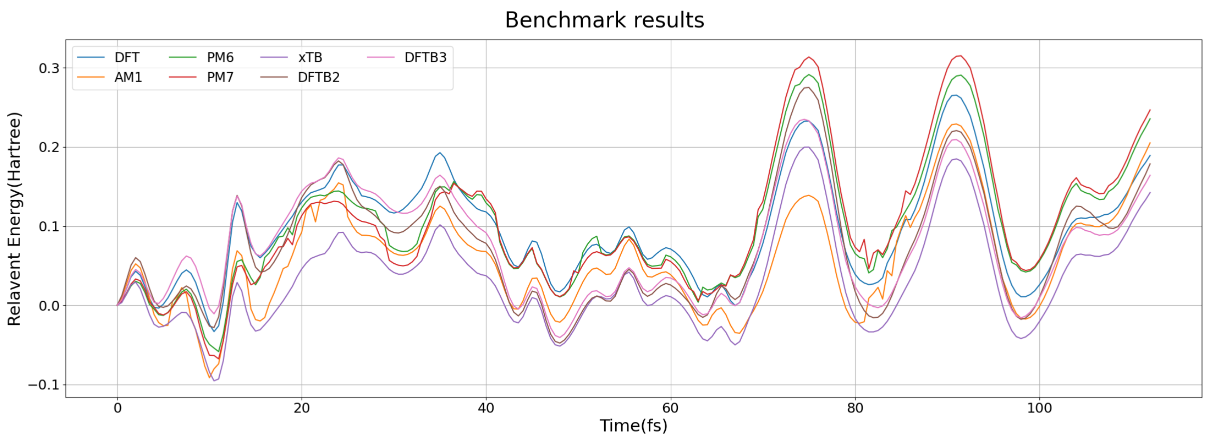

Figure S77: Carbon-19,Trajectory-2.

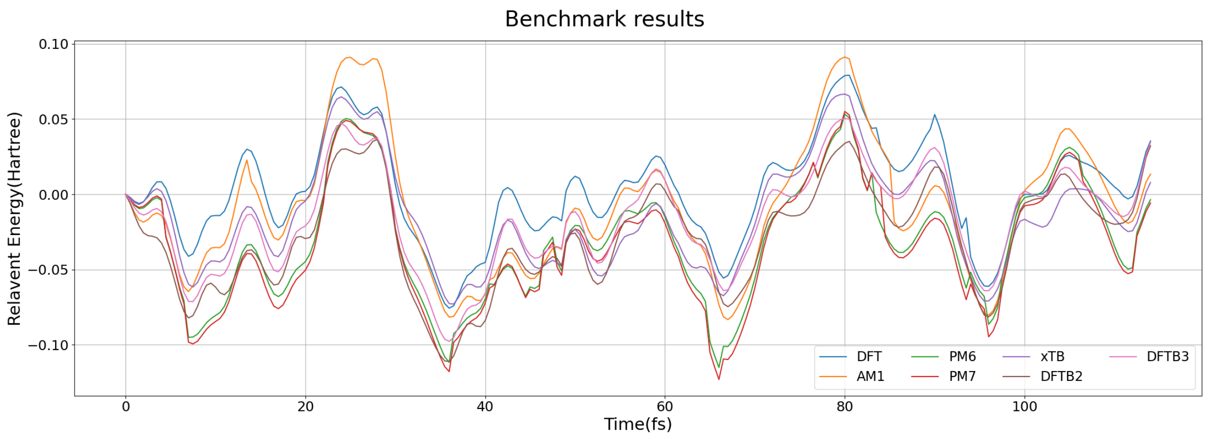

Figure S78: Carbon-19,Trajectory-3.

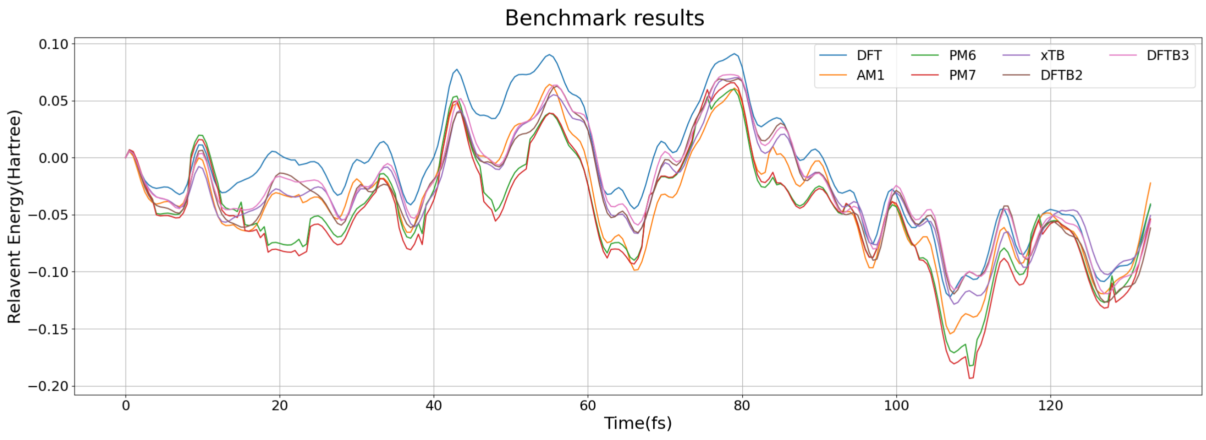

Figure S79: Carbon-19,Trajectory-4.

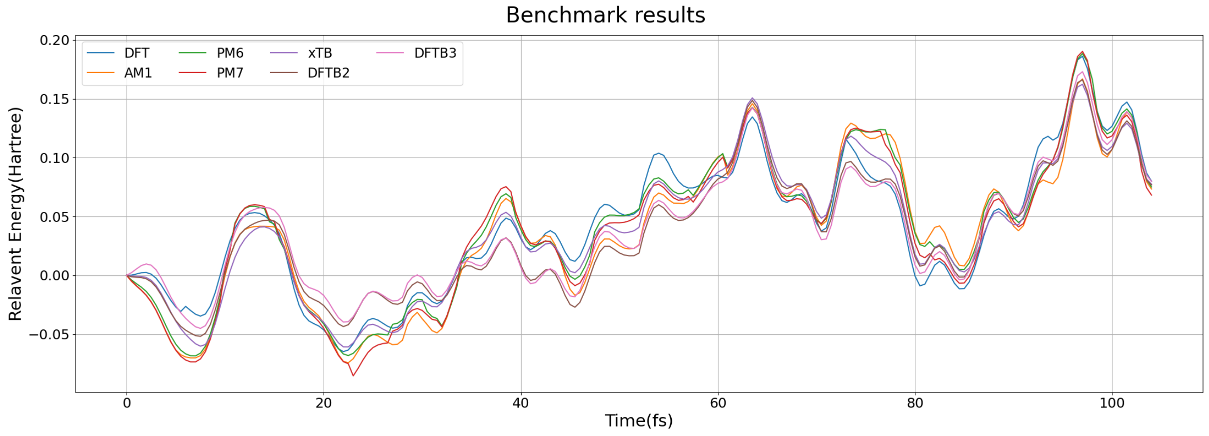

Figure S80: Carbon-19,Trajectory-5.

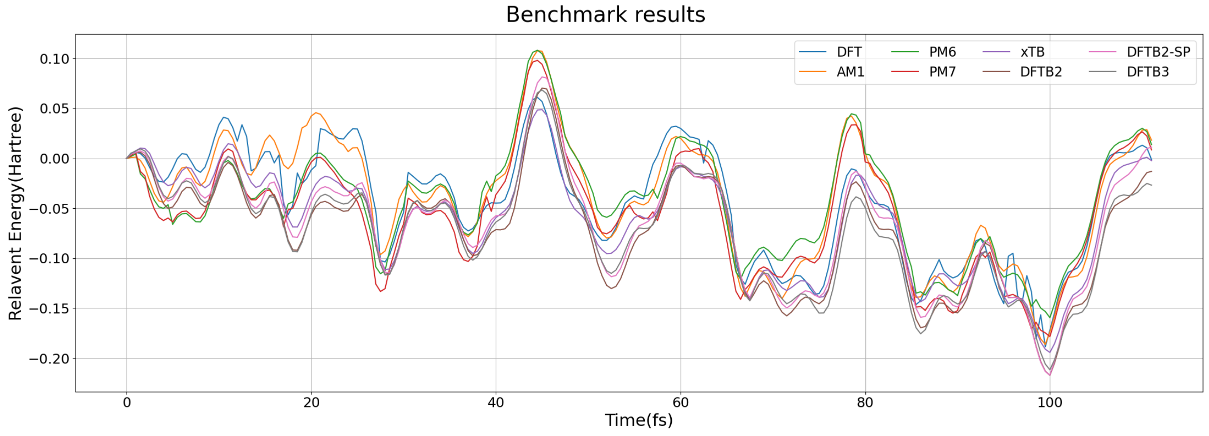

Figure S81: Carbon-20,Trajectory-1.

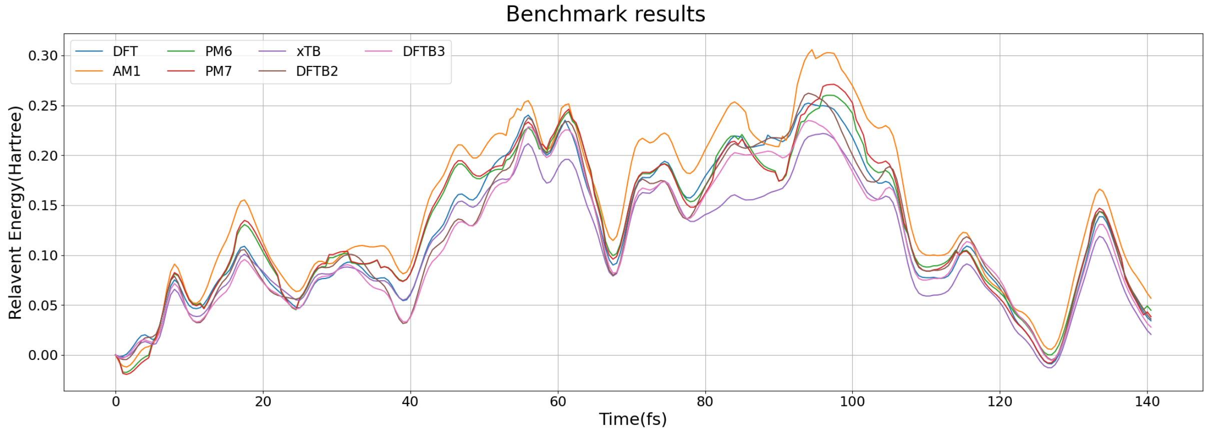

Figure S82: Carbon-20,Trajectory-2.

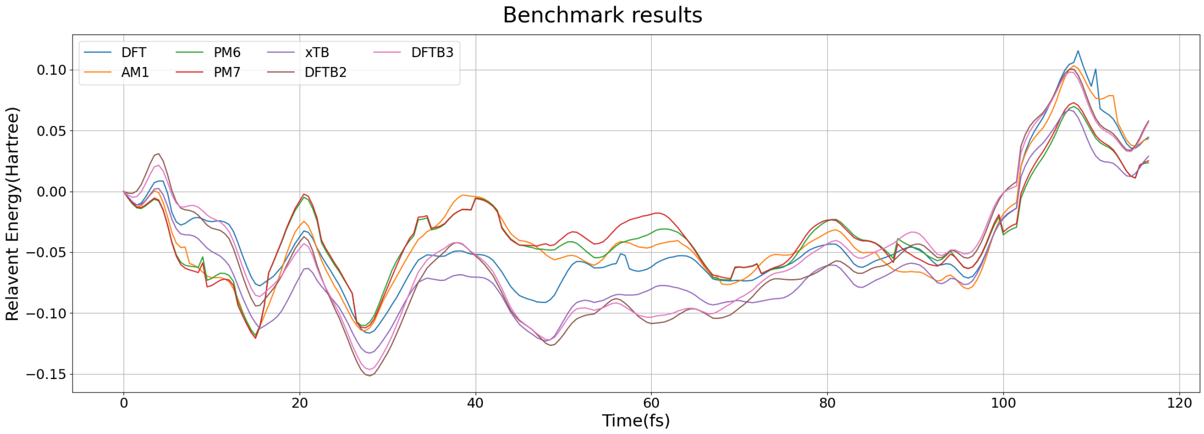

Figure S83: Carbon-23,Trajectory-1.

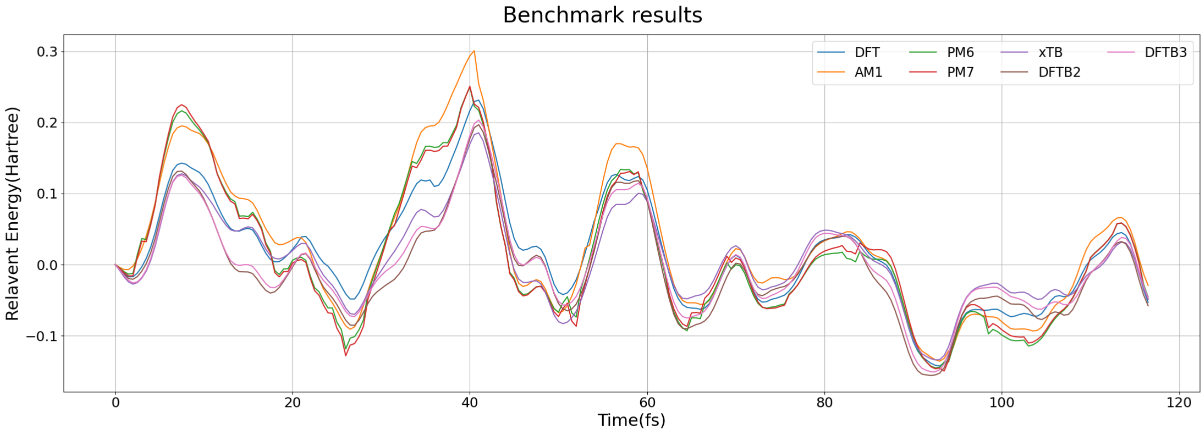

Figure S84: Carbon-24,Trajectory-1.

2 Trajectory energy profiles shape

In the article, Spearman's rank correlation coefficient ρ is used to quantitatively measure the similarity of energy profiles, this coefficient is calculated by Equation 1,

$$\rho = 1 - \frac{6\sum d_i^2}{n(n^2-1)}. \tag{1}$$

For the 84 trajectories, the corresponding DFT energy profile is used as a reference, and the coefficient ρ of each SE method is calculated respectively. All the data are listed in Table. S1 and Table. S2, and the colorscale plot Fig. S85 and Fig. S86 containing the data is also provided, the rightmost side of each row is the average value of the trajectory energy profile ρ value of the soot molecule calculated by all methods. The end of Fig. S86 shows the average of all soot molecular coefficient ρ values calculated for each method, the colorscale plot is used to represent the the overall performance of the different SE methods. The data in the table is the value of ρ, the values are the same as that in Table. S1 and Table. S2.

	AM1	PM6	PM7	xTB	DFTB2-SP	DFTB2	DFTB3	**AVERAGE**
C4-1	0.9496	0.9438	0.9411	0.9631	0.8909	0.8820	0.9165	**0.9267**
C4-2	0.9418	0.9426	0.9474	0.9526	-	0.8855	0.9156	**0.9309**
C5-1	0.9717	0.9431	0.9415	0.9036	0.9435	0.9635	0.9774	**0.9492**
C5-2	0.7939	0.8132	0.7912	0.9138	-	0.8995	0.9642	**0.8626**
C5-3	0.9454	0.9194	0.9028	0.8127	-	0.9044	0.8752	**0.8933**
C5-4	0.9443	0.9002	0.9071	0.9818	-	0.9539	0.9469	**0.9390**
C5-5	0.9474	0.8646	0.8674	0.9816	-	0.9576	0.9715	**0.9317**
C5-6	0.9661	0.9694	0.9729	0.9781	-	0.9745	0.9688	**0.9716**
C5-7	0.9701	0.8518	0.8570	0.9561	-	0.9591	0.9503	**0.9241**
C6-1	0.9243	0.9332	0.9472	0.9728	0.9545	0.9451	0.9564	**0.9476**
C6-2	0.9639	0.9442	0.9330	0.9467	0.9139	0.9123	0.9421	**0.9366**
C6-3	0.9631	0.9663	0.9676	0.9702	-	0.9757	0.9639	**0.9678**
C6-4	0.8951	0.9085	0.8965	0.9613	0.9151	0.9187	0.9475	**0.9204**
C6-5	0.8476	0.8628	0.8141	0.9055	-	0.8262	0.8811	**0.8562**
C6-6	0.9629	0.9088	0.9063	0.9250	-	0.9213	0.9446	**0.9282**
C6-7	0.9785	0.9425	0.9331	0.9736	-	0.9621	0.9760	**0.9610**
C6-8	0.9394	0.8644	0.8271	0.9249	-	0.9111	0.9534	**0.9034**
C6-9	0.9642	0.9462	0.9330	0.9751	-	0.9772	0.9754	**0.9618**
C6-10	0.9339	0.8508	0.8390	0.9421	0.9051	0.8922	0.9510	**0.9020**
C6-11	0.8901	0.8834	0.8837	0.9094	0.9032	0.8990	0.9008	**0.8957**
C6-12	0.9532	0.8949	0.8658	0.9601	-	0.9623	0.9614	**0.9330**
C6-13	0.9615	0.9488	0.9542	0.9860	-	0.9687	0.9638	**0.9638**
C6-14	0.9277	0.9464	0.9316	0.9634	-	0.9302	0.9827	**0.9470**
C6-15	0.9499	0.9464	0.9238	0.6468	-	0.8245	0.7939	**0.8475**
C6-16	0.9201	0.9301	0.9101	0.9739	-	0.8922	0.9379	**0.9274**
C6-17	0.9245	0.9121	0.8997	0.9216	-	0.8701	0.8902	**0.9030**
C6-18	0.7667	0.7715	0.7570	0.7335	-	0.8728	0.9162	**0.8030**
C7-1	0.9515	0.9690	0.9675	0.9792	0.9861	0.9856	0.9763	**0.9736**
C7-2	0.9504	0.9394	0.9342	0.8743	-	0.9443	0.8581	**0.9168**
C7-3	0.8801	0.8696	0.8572	0.9357	0.8767	0.8820	0.9533	**0.8935**
C7-4	0.9819	0.9756	0.9734	0.9896	-	0.9646	0.9535	**0.9731**
C7-5	0.9253	0.8876	0.8892	0.9559	-	0.9582	0.9676	**0.9306**
C7-6	0.9687	0.9600	0.9603	0.9895	-	0.9829	0.9862	**0.9746**
C7-7	0.8921	0.9077	0.9071	0.9792	-	0.9510	0.9517	**0.9315**
C7-8	0.9193	0.8691	0.8699	0.9680	0.9361	0.9121	0.9449	**0.9171**
C7-9	0.8513	0.8996	0.8944	0.9934	0.9264	0.9235	0.9548	**0.9205**
C8-1	0.9472	0.9703	0.9599	0.9034	-	0.9195	0.9131	**0.9356**
C8-2	0.6844	0.7473	0.7074	0.9077	0.8746	0.8771	0.9326	**0.8187**
C8-3	0.9098	0.9298	0.9358	0.9660	0.9228	0.9221	0.9263	**0.9304**
C8-4	0.9591	0.9393	0.9257	0.9814	-	0.9398	0.9474	**0.9488**
C8-5	0.9497	0.9528	0.9529	0.9548	-	0.8985	0.9348	**0.9406**
C8-6	0.9292	0.9199	0.9165	0.9477	0.9583	0.9532	0.9460	**0.9387**
C8-7	0.7233	0.8548	0.8142	0.9459	0.7244	0.7294	0.8697	**0.8088**

Figure S85: The rules of the colorscale plot in the figure are the same as in the article, the larger the ρ, the trajectory energy calculated by the corresponding SE method is closer to the calculation result of the DFT method , then it corresponds to darker green, and the smaller ρ is, the closer the color is to dark red. The rightmost column of the table is the average of the ρ values of all SE methods corresponding to each track, and this table is the Part-1.

	AM1	PM6	PM7	xTB	DFTB2-SP	DFTB2	DFTB3	**AVERAGE**
C10-1	0.8830	0.8989	0.8548	0.9193	-	0.8176	0.8904	**0.8773**
C10-2	0.9233	0.9276	0.8996	0.9496	-	0.9215	0.9584	**0.9300**
C10-3	0.7492	0.8054	0.7767	0.8787	-	0.8795	0.9257	**0.8359**
C10-4	0.9134	0.8015	0.7940	0.9453	-	0.9071	0.9136	**0.8791**
C10-5	0.8710	0.8087	0.7925	0.8551	-	0.8773	0.8938	**0.8497**
C10-6	0.9513	0.8726	0.8694	0.9287	-	0.8935	0.9152	**0.9051**
C10-7-O	0.9703	0.9653	0.9708	0.9830	-	0.8832	0.8792	**0.9420**
C11-1	0.8234	0.8200	0.8190	0.7807	-	0.8384	0.8448	**0.8210**
C11-2	0.9513	0.8873	0.8753	0.9591	-	0.9654	0.9792	**0.9363**
C11-3	0.9563	0.9650	0.9644	0.9879	0.9568	0.9603	0.9685	**0.9656**
C11-4	0.8850	0.8740	0.8655	0.9464	-	0.8908	0.9276	**0.8982**
C11-5	0.9753	0.9707	0.9269	0.9833	-	0.9496	0.9593	**0.9609**
C12-1	0.9411	0.9462	0.9398	0.9550	-	0.8867	0.8876	**0.9261**
C12-2-O	0.9713	0.9366	0.9450	0.9819	-	0.9676	0.9647	**0.9612**
C12-3	0.8341	0.8747	0.8198	0.9050	-	0.7726	0.8503	**0.8427**
C12-4	0.9796	0.9744	0.9722	0.9534	-	0.9290	0.9468	**0.9592**
C12-5	0.9034	0.9032	0.9025	0.9281	-	0.9535	0.9534	**0.9240**
C12-6	0.9520	0.8526	0.8336	0.9057	-	0.8829	0.8631	**0.8817**
C12-7	0.8969	0.8440	0.8639	0.9853	0.9354	0.9384	0.9794	**0.9205**
C14-1	0.8944	0.9149	0.9176	0.9746	-	0.9688	0.9794	**0.9416**
C15-1	0.9448	0.9237	0.9142	0.9861	-	0.9611	0.9827	**0.9521**
C16-1	0.9480	0.9428	0.9426	0.9879	0.9779	0.9711	0.9821	**0.9646**
C16-2	0.9430	0.9062	0.8798	0.9549	-	0.9372	0.9507	**0.9286**
C16-3	0.9625	0.9425	0.9360	0.9491	-	0.9572	0.9386	**0.9477**
C16-4	0.9341	0.9243	0.9256	0.9784	-	0.9516	0.9538	**0.9446**
C16-5	0.9574	0.9532	0.9532	0.9835	0.9646	0.9468	0.9620	**0.9601**
C17-1	0.9342	0.9139	0.9100	0.9645	-	0.9486	0.9636	**0.9391**
C17-2	0.9655	0.9650	0.9612	0.9765	-	0.9870	0.9923	**0.9746**
C17-3	0.9502	0.9389	0.9241	0.9743	-	0.9317	0.9311	**0.9417**
C17-4-O	0.9784	0.9273	0.9375	0.9735	-	0.9932	0.9954	**0.9675**
C17-5	0.8631	0.8370	0.7900	0.9003	-	0.9459	0.9622	**0.8831**
C17-6	0.8467	0.9194	0.9206	0.9882	0.9252	0.9211	0.9651	**0.9266**
C19-1	0.9352	0.9121	0.9108	0.9767	-	0.8872	0.9327	**0.9258**
C19-2	0.9294	0.9080	0.8591	0.9456	-	0.9187	0.9272	**0.9146**
C19-3	0.9179	0.8625	0.8700	0.9225	-	0.8975	0.9439	**0.9024**
C19-4	0.9563	0.8928	0.8771	0.9750	-	0.9665	0.9808	**0.9414**
C19-5	0.9376	0.9673	0.9634	0.9747	-	0.9457	0.9536	**0.9570**
C20-1	0.8991	0.8217	0.8538	0.9484	0.9385	0.9289	0.9408	**0.9045**
C20-2	0.9793	0.9815	0.9774	0.9814	-	0.9884	0.9890	**0.9828**
C23-1	0.7008	0.6998	0.6620	0.9468	-	0.8983	0.9017	**0.8016**
C24-1	0.9548	0.9489	0.9371	0.9338	-	0.9343	0.9485	**0.9429**
	**AM1**	**PM6**	**PM7**	**xTB**	**DFTB2-SP**	**DFTB2**	**DFTB3**	
**AVERAGE**	0.9220	0.9043	0.8948	0.9521	0.9497	0.9273	0.9391	**0.9319**

Figure S86: Colorscale plot of the 84 trajectories, the bottom of the table is the average value of ρ obtained by calculating 84 trajectories by the SE method corresponding to this column, this table is the Part-2.

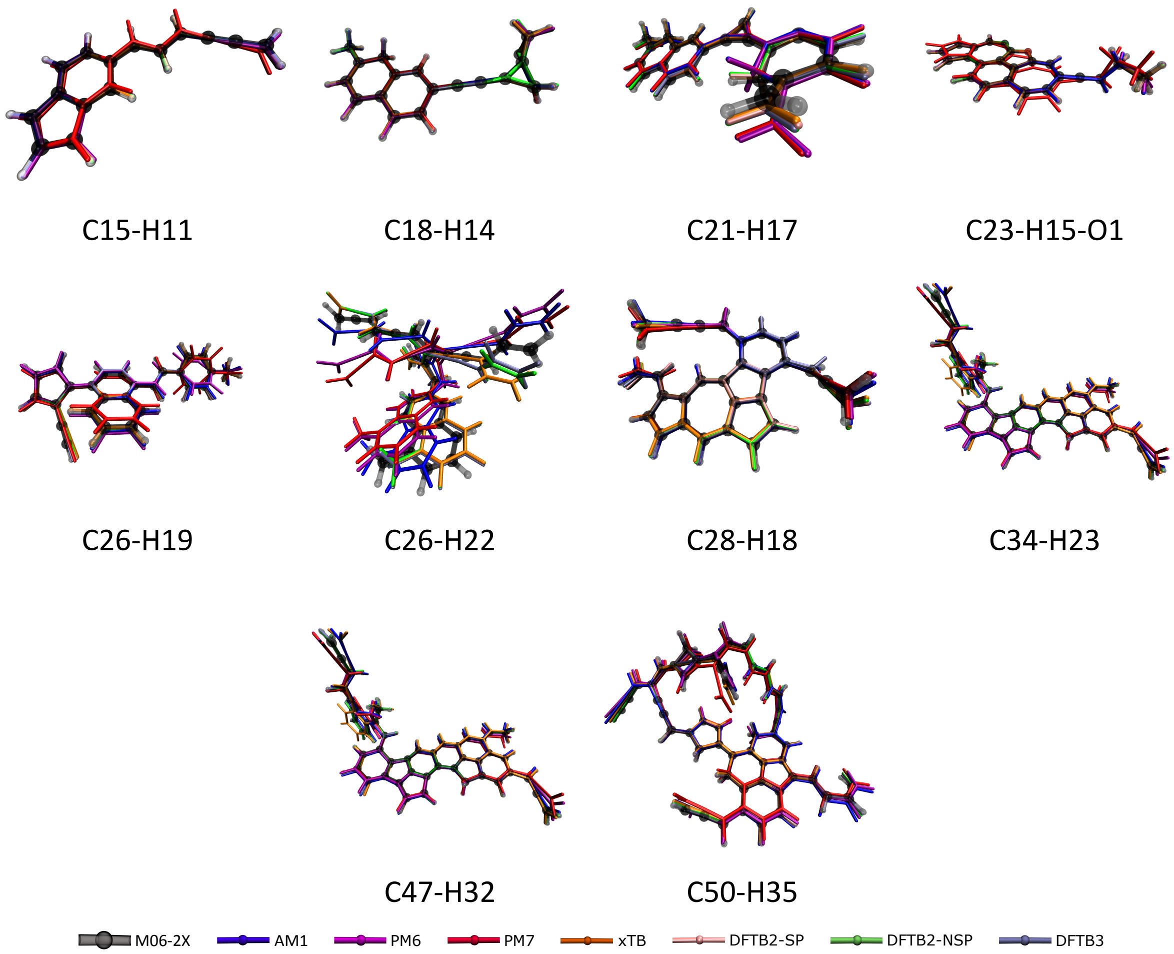

Figure S87: In order to verify the performance of the various methods in the previous section, 10 soot molecules in MD trajectory are selected as examples, The figure shows the structures of 10 soot molecules optimized by different SE methods, of which the semitransparent structure is the reference optimized by the DFT method, and the structures optimized by other SE methods are represented by lines with different colors.

3 The structures optimized by different methods

In order to verify the performance of the various methods in the previous section, 10 soot molecules in MD trajectory are selected as examples, in the article, we only show the structural comparison of C50-H35 molecule optimized by different methods as an example. In the Supporting Information part, the structural comparison of all molecules will be shown in Fig. S87. Table. S3 is the same as Table. 1 in the article, these can be used for structural comparisons of other 9 molecules.

In the article, in addition to the 10 soot molecules selected from the molecular dynamics trajectories, we also selected a group of 33 molecules from the RMG database to test the optimized structures of the SE methods in the text. All the data and structure are shown in Table. S4 and Table. S5.

Table S1: 84 MD trajectories from MD simulation were used to validate the accuracy of SE methods against M06-2x/def2TZVPP level QM calculation.The table lists the coefficient ρ values of the energy profiles calculated by different SE methods for each trajectory compared with the DFT calculation results. ρ is calculated according to Equation. 1 in the article, and this Table is the Part-1.

	AM1	PM6	PM7	xTB	DFTB2-SP	DFTB2	DFTB3
C4-1	0.9496	0.9438	0.9411	0.9631	0.8909	0.8820	0.9165
C4-2	0.9418	0.9426	0.9474	0.9526	-	0.8855	0.9156
C5-1	0.9717	0.9431	0.9415	0.9036	0.9435	0.9635	0.9774
C5-2	0.7939	0.8132	0.7912	0.9138	-	0.8995	0.9642
C5-3	0.9454	0.9194	0.9028	0.8127	-	0.9044	0.8752
C5-4	0.9443	0.9002	0.9071	0.9818	-	0.9539	0.9469
C5-5	0.9474	0.8646	0.8674	0.9816	-	0.9576	0.9715
C5-6	0.9661	0.9694	0.9729	0.9781	-	0.9745	0.9688
C5-7	0.9701	0.8518	0.8570	0.9561	-	0.9591	0.9503
C6-1	0.9243	0.9332	0.9472	0.9728	0.9545	0.9451	0.9564
C6-2	0.9639	0.9442	0.9330	0.9467	0.9139	0.9123	0.9421
C6-3	0.9631	0.9663	0.9676	0.9702	-	0.9757	0.9639
C6-4	0.8951	0.9085	0.8965	0.9613	0.9151	0.9187	0.9475
C6-5	0.8476	0.8628	0.8141	0.9055	-	0.8262	0.8811
C6-6	0.9629	0.9088	0.9063	0.9250	-	0.9213	0.9446
C6-7	0.9785	0.9425	0.9331	0.9736	-	0.9621	0.9760
C6-8	0.9394	0.8644	0.8271	0.9249	-	0.9111	0.9534
C6-9	0.9642	0.9462	0.9330	0.9751	-	0.9772	0.9754
C6-10	0.9339	0.8508	0.8390	0.9421	0.9051	0.8922	0.9510
C6-11	0.8901	0.8834	0.8837	0.9094	0.9032	0.8990	0.9008
C6-12	0.9532	0.8949	0.8658	0.9601	-	0.9623	0.9614
C6-13	0.9615	0.9488	0.9542	0.9860	-	0.9687	0.9638
C6-14	0.9277	0.9464	0.9316	0.9634	-	0.9302	0.9827
C6-15	0.9499	0.9464	0.9238	0.6468	-	0.8245	0.7939
C6-16	0.9201	0.9301	0.9101	0.9739	-	0.8922	0.9379
C6-17	0.9245	0.9121	0.8997	0.9216	-	0.8701	0.8902
C6-18	0.7667	0.7715	0.7570	0.7335	-	0.8728	0.9162
C7-1	0.9515	0.9690	0.9675	0.9792	0.9861	0.9856	0.9763
C7-2	0.9504	0.9394	0.9342	0.8743	-	0.9443	0.8581
C7-3	0.8801	0.8696	0.8572	0.9357	0.8767	0.8820	0.9533
C7-4	0.9819	0.9756	0.9734	0.9896	-	0.9646	0.9535
C7-5	0.9253	0.8876	0.8892	0.9559	-	0.9582	0.9676
C7-6	0.9687	0.9600	0.9603	0.9895	-	0.9829	0.9862
C7-7	0.8921	0.9077	0.9071	0.9792	-	0.9510	0.9517
C7-8	0.9193	0.8691	0.8699	0.9680	0.9361	0.9121	0.9449
C7-9	0.8513	0.8996	0.8944	0.9934	0.9264	0.9235	0.9548
C8-1	0.9472	0.9703	0.9599	0.9034	-	0.9195	0.9131
C8-2	0.6844	0.7473	0.7074	0.9077	0.8746	0.8771	0.9326
C8-3	0.9098	0.9298	0.9358	0.9660	0.9228	0.9221	0.9263
C8-4	0.9591	0.9393	0.9257	0.9814	-	0.9398	0.9474
C8-5	0.9497	0.9528	0.9529	0.9548	-	0.8985	0.9348
C8-6	0.9292	0.9199	0.9165	0.9477	0.9583	0.9532	0.9460
C8-7	0.7233	0.8548	0.8142	0.9459	0.7244	0.7294	0.8697

Table S2: The table lists the coefficient ρ values of the energy profiles calculated by different SE methods for each trajectory compared with the DFT calculation results, this is the Part-2.

	AM1	PM6	PM7	xTB	DFTB2-SP	DFTB2	DFTB3
C10-1	0.8830	0.8989	0.8548	0.9193	-	0.8176	0.8904
C10-2	0.9233	0.9276	0.8996	0.9496	-	0.9215	0.9584
C10-3	0.7492	0.8054	0.7767	0.8787	-	0.8795	0.9257
C10-4	0.9134	0.8015	0.7940	0.9453	-	0.9071	0.9136
C10-5	0.8710	0.8087	0.7925	0.8551	-	0.8773	0.8938
C10-6	0.9513	0.8726	0.8694	0.9287	-	0.8935	0.9152
C10-7-O	0.9703	0.9653	0.9708	0.9830	-	0.8832	0.8792
C11-1	0.8234	0.8200	0.8190	0.7807	-	0.8384	0.8448
C11-2	0.9513	0.8873	0.8753	0.9591	-	0.9654	0.9792
C11-3	0.9563	0.9650	0.9644	0.9879	0.9568	0.9603	0.9685
C11-4	0.8850	0.8740	0.8655	0.9464	-	0.8908	0.9276
C11-5	0.9753	0.9707	0.9269	0.9833	-	0.9496	0.9593
C12-1	0.9411	0.9462	0.9398	0.9550	-	0.8867	0.8876
C12-2-O	0.9713	0.9366	0.9450	0.9819	-	0.9676	0.9647
C12-3	0.8341	0.8747	0.8198	0.9050	-	0.7726	0.8503
C12-4	0.9796	0.9744	0.9722	0.9534	-	0.9290	0.9468
C12-5	0.9034	0.9032	0.9025	0.9281	-	0.9535	0.9534
C12-6	0.9520	0.8526	0.8336	0.9057	-	0.8829	0.8631
C12-7	0.8969	0.8440	0.8639	0.9853	0.9354	0.9384	0.9794
C14-1	0.8944	0.9149	0.9176	0.9746	-	0.9688	0.9794
C15-1	0.9448	0.9237	0.9142	0.9861	-	0.9611	0.9827
C16-1	0.9480	0.9428	0.9426	0.9879	0.9779	0.9711	0.9821
C16-2	0.9430	0.9062	0.8798	0.9549	-	0.9372	0.9507
C16-3	0.9625	0.9425	0.9360	0.9491	-	0.9572	0.9386
C16-4	0.9341	0.9243	0.9256	0.9784	-	0.9516	0.9538
C16-5	0.9574	0.9532	0.9532	0.9835	0.9646	0.9468	0.9620
C17-1	0.9342	0.9139	0.9100	0.9645	-	0.9486	0.9636
C17-2	0.9655	0.9650	0.9612	0.9765	-	0.9870	0.9923
C17-3	0.9502	0.9389	0.9241	0.9743	-	0.9317	0.9311
C17-4-O	0.9784	0.9273	0.9375	0.9735	-	0.9932	0.9954
C17-5	0.8631	0.8370	0.7900	0.9003	-	0.9459	0.9622
C17-6	0.8467	0.9194	0.9206	0.9882	0.9252	0.9211	0.9651
C19-1	0.9352	0.9121	0.9108	0.9767	-	0.8872	0.9327
C19-2	0.9294	0.9080	0.8591	0.9456	-	0.9187	0.9272
C19-3	0.9179	0.8625	0.8700	0.9225	-	0.8975	0.9439
C19-4	0.9563	0.8928	0.8771	0.9750	-	0.9665	0.9808
C19-5	0.9376	0.9673	0.9634	0.9747	-	0.9457	0.9536
C20-1	0.8991	0.8217	0.8538	0.9484	0.9385	0.9289	0.9408
C20-2	0.9793	0.9815	0.9774	0.9814	-	0.9884	0.9890
C23-1	0.7008	0.6998	0.6620	0.9468	-	0.8983	0.9017
C24-1	0.9548	0.9489	0.9371	0.9338	-	0.9343	0.9485

Table S3: In Fig. S87, 10 soot molecules in MD trajectory are selected as examples, The RMSE of the structures optimized by each SE method and the DFT reference structure were calculated, the results were shown in this table.

	AM1	PM6	PM7	xTB	DFTB2-SP	DFTB2	DFTB3
C15-H11	0.0456	0.0915	0.3517	0.0563	0.0602	0.0602	0.0536
C18-H14	0.0824	0.1040	0.0943	0.0674	-	0.0887	0.0761
C21-H17	0.5073	0.5068	0.5143	0.1463	0.1689	0.2087	0.2087
C23-H15-O1	0.1819	0.1710	0.5080	0.0497	0.0642	0.0609	0.0656
C26-H19	0.3568	0.6439	0.6862	0.1690	0.1931	0.1929	0.2592
C26-H22	1.2711	2.4898	2.5142	0.9765	-	0.7924	0.7377
C28-H18	0.2743	0.2878	0.2850	0.3956	0.3964	0.3883	0.3957
C34-H23	0.7308	0.8423	0.8707	0.2865	0.2803	0.2870	0.2595
C47-H32	0.3241	0.5465	0.6677	0.4234	-	0.0998	0.1037
C50-H35	0.3511	0.4144	0.5725	0.2471	0.1177	0.1183	0.0671

Table S4: A group of 33 molecules selected from the RMG database were optimized by the DFT method and various SE methods in the Table, respectively, then taking the DFT method as a reference, the RMSEs of the structures optimized by various SE methods and the reference structures were calculated, this Table is the Part-1.

	Structure	AM1	PM6	PM7	xTB	DFTB2-SP	DFTB2	DFTB3
Soot 1	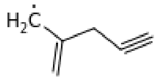	0.0241	0.0249	0.0324	0.0212	0.0216	0.0237	0.0126
Soot 2	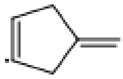	0.0252	0.0285	0.0271	0.0131	0.0213	0.0141	0.0166
Soot 3	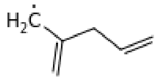	0.6965	0.3105	0.4668	0.0304	0.0149	0.0141	0.0104
Soot 4	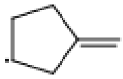	0.0827	0.0813	0.0814	0.0584	0.0562	0.0602	0.0517
Soot 5	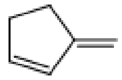	0.0827	0.0813	0.0813	0.0584	0.0639	0.0547	0.0527
Soot 6	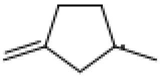	0.4062	0.4082	0.4096	0.1022	0.2345	0.2264	0.1892
Soot 7	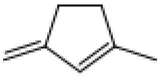	0.0746	0.0731	0.0723	0.0506	0.0506	0.0251	0.0350
Soot 8	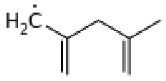	0.4908	0.1916	0.2807	0.0935	0.0142	0.0120	0.0139
Soot 9	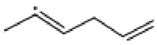	0.4381	0.5875	0.5288	0.0579	0.0479	0.0184	0.0269
Soot 10	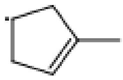	0.0341	0.0264	0.0262	0.0145	0.0155	0.0141	0.0130
Soot 11	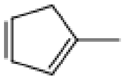	0.0315	0.0257	0.0251	0.0161	0.0349	0.0134	0.0107
Soot 12	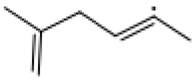	0.6517	0.6197	0.5414	0.1468	0.0493	0.0327	0.0237
Soot 13	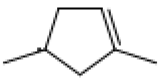	0.0637	0.0500	0.0507	0.0271	0.0320	0.0299	0.0308
Soot 14	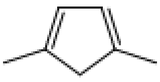	0.0430	0.0308	0.0307	0.0199	0.0357	0.0164	0.0138
Soot 15	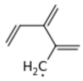	0.1948	0.3434	0.4015	0.0292	0.1237	0.1001	0.0576
Soot 16	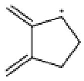	0.2674	0.2678	0.2672	0.2046	0.1626	0.1502	0.1549

Table S5: RMSE of 33 Optimized geometry of soots, Part-2

	Structure	AM1	PM6	PM7	xTB	DFTB2-SP	DFTB2	DFTB3
Soot 17	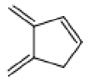	0.0253	0.0277	0.0265	0.0130	0.0295	0.0175	0.0126
Soot 18	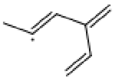	0.3045	1.2356	1.2093	0.1694	0.0784	0.1245	0.0463
Soot 19	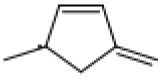	0.0315	0.0271	0.0257	0.0121	0.0217	0.0238	0.0415
Soot 20	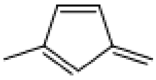	0.0273	0.0281	0.0260	0.0139	0.0359	0.0162	0.0183
Soot 21	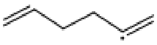	0.5905	0.3708	0.3567	0.0812	0.0323	0.0221	0.0129
Soot 22	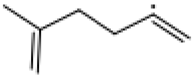	0.3145	0.3656	0.3603	0.0575	0.0278	0.0195	0.0147
Soot 23	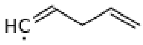	0.2907	0.2522	0.3126	0.0361	0.0438	0.0336	0.0441
Soot 24	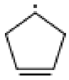	0.4667	0.3288	0.2439	0.0248	0.1274	0.1195	0.1332
Soot 25	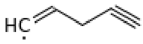	0.1812	0.3018	0.2288	0.0523	0.0517	0.0343	0.0337
Soot 26	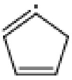	0.1506	0.1231	0.1249	0.0964	0.1885	0.1066	0.1861
Soot 27	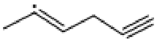	0.3013	0.4114	0.3680	0.0624	0.0600	0.0289	0.0402
Soot 28	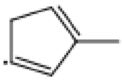	0.0316	0.0313	0.0299	0.0180	0.0216	0.0145	0.0180
Soot 29	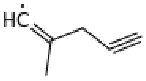	0.2191	0.1891	0.2125	0.1352	0.0515	0.0361	0.0416
Soot 30	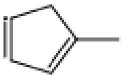	0.0377	0.0339	0.0326	0.0162	0.0249	0.0169	0.0215
Soot 31	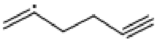	0.3323	0.6863	0.5242	0.0424	0.0337	0.0228	0.0272
Soot 32	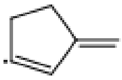	0.0549	0.0553	0.0550	0.0169	0.0258	0.0168	0.0211
Soot 33	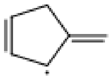	0.0258	0.0227	0.0205	0.0116	0.0175	0.0151	0.0101
